# Identifying health risk determinants and molecular targets in patients with idiopathic pulmonary fibrosis via combined differential and weighted gene co-expression analysis

**DOI:** 10.3389/fgene.2024.1496462

**Published:** 2025-01-29

**Authors:** Abu Tayab Moin, Md. Asad Ullah, Jannatul Ferdous Nipa, Mohammad Sheikh Farider Rahman, Afsana Emran, Md. Minhazul Islam, Swapnil Das, Tawsif Al Arian, Mohammad Mahfuz Enam Elahi, Mukta Akter, Umme Sadea Rahman, Arnab Halder, Shoaib Saikat, Mohammad Jakir Hosen

**Affiliations:** ^1^ Laboratory of Clinical Genetics, Genomics and Enzyme Research, Department of Genetic Engineering and Biotechnology, Faculty of Biological Sciences, University of Chittagong, Chattogram, Bangladesh; ^2^ Department of Biotechnology and Genetic Engineering, Faculty of Biological Sciences, Jahangirnagar University, Dhaka, Bangladesh; ^3^ Department of Genetic Engineering and Biotechnology, East West University, Dhaka, Bangladesh; ^4^ Department of Molecular Biotechnology, Applied Bioscience and Process Engineering, Anhalt University of Applied Sciences, Köthen, Germany; ^5^ Department of Pharmacy, BGC Trust University Bangladesh, Chattogram, Bangladesh; ^6^ Department of Pharmacy, University of Science and Technology Chittagong (USTC), Chattogram, Bangladesh; ^7^ Department of Pharmacy, Faculty of Biological Science, Jahangirnagar University, Dhaka, Savar, Bangladesh; ^8^ Department of Pharmacy, University of Asia Pacific, Dhaka, Bangladesh; ^9^ Department of Agricultural Extension, Ministry of Agriculture, Dhaka, Bangladesh; ^10^ Department of Pharmacy, Independent University, Dhaka, Bangladesh; ^11^ Department of Biochemistry and Biotechnology, Faculty of Bio-Sciences, University of Barishal, Barishal, Bangladesh; ^12^ Department of Genetic Engineering and Biotechnology, School of Life Sciences, Shahjalal University of Science and Technology, Sylhet, Bangladesh

**Keywords:** idiopathic pulmonary fibrosis, transcriptome analysis, differentially expressed genes, lung tissue, drug targets, biomarkers, molecular mechanisms, pulmonary disorders

## Abstract

**Introduction:**

Idiopathic pulmonary fibrosis (IPF) is a rare but debilitating lung disease characterized by excessive fibrotic tissue accumulation, primarily affecting individuals over 50 years of age. Early diagnosis is challenging, and without intervention, the prognosis remains poor. Understanding the molecular mechanisms underlying IPF pathogenesis is crucial for identifying diagnostic markers and therapeutic targets.

**Methods:**

We analyzed transcriptomic data from lung tissues of IPF patients using two independent datasets. Differentially expressed genes (DEGs) were identified, and their functional roles were assessed through pathway enrichment and tissue-specific expression analysis. Protein-protein interaction (PPI) networks and co-expression modules were constructed to identify hub genes and their associations with disease severity. Machine learning approaches were applied to identify genes capable of differentiating IPF patients from healthy individuals. Regulatory signatures, including transcription factor and microRNA interactions, were also explored, alongside the identification of potential drug targets.

**Results:**

A total of 275 and 167 DEGs were identified across two datasets, with 67 DEGs common to both. These genes exhibited distinct expression patterns across tissues and were associated with pathways such as extracellular matrix organization, collagen fibril formation, and cell adhesion. Co-expression analysis revealed DEG modules correlated with varying IPF severity phenotypes. Machine learning analysis pinpointed a subset of genes with high discriminatory power between IPF and healthy individuals. PPI network analysis identified hub proteins involved in key biological processes, while functional enrichment reinforced their roles in extracellular matrix regulation. Regulatory analysis highlighted interactions with transcription factors and microRNAs, suggesting potential mechanisms driving IPF pathogenesis. Potential drug targets among the DEGs were also identified.

**Discussion:**

This study provides a comprehensive transcriptomic overview of IPF, uncovering DEGs, hub proteins, and regulatory signatures implicated in disease progression. Validation in independent datasets confirmed the relevance of these findings. The insights gained here lay the groundwork for developing diagnostic tools and novel therapeutic strategies for IPF.

## 1 Introduction

Idiopathic pulmonary fibrosis (IPF) is a chronic, progressive lung disease characterized by the buildup of fibrotic tissue within the lung parenchyma, leading to severe impairments in gas exchange, respiratory failure, and ultimately poor patient outcomes (Schwartz, 2016). This abnormal accumulation of the extracellular matrix (ECM) disrupts alveolar function and results in reduced lung compliance (Richeldi et al., 2017). Although environmental factors (such as wood dust, silica, and microbial agents like viruses, fungi, and bacteria) and genetic and epigenetic predispositions contribute to IPF pathogenesis, the precise molecular drivers of this complex condition remain incompletely understood. In particular, aging, smoking, and certain gene expression changes have been identified as key risk factors; however, the specific biological pathways that drive fibrosis initiation and progression are not fully elucidated (Issa, 2014; Liu et al., 2010).

Although IPF is classified as a rare disease, its prevalence ranges from 0.33 to 4.51 per 10,000 individuals globally, with an estimated 30,000 to 40,000 new cases annually (Maher et al., 2021; Srour et al., 2017). IPF predominantly affects individuals over the age of 50, with a mean age of diagnosis between 65 and 70 years, and the disease progresses rapidly without effective intervention, with a typical survival rate of only 2–3 years post-diagnosis (Fernández-Fabrellas et al., 2019; Collard, 2010; Sharif, 2017). Current diagnostic approaches rely heavily on imaging and, in some cases, invasive surgical lung biopsies (SLBs) (Liao et al., 2023). Therapeutic options are limited to anti-fibrotic agents like nintedanib and pirfenidone, which carry significant side effects and do not prevent disease progression (Kang and Song, 2021). As a result, there is an urgent need to identify more precise biomarkers for diagnosis and develop novel therapeutic strategies that target key pathways involved in fibrosis.

A critical gap in IPF research lies in the comprehensive understanding of the transcriptomic alterations within the lung tissues of IPF patients. Previous studies employing transcriptomic analyses have primarily identified differential gene expression and dysregulated pathways associated with fibrosis, yet they often do not address comorbidities and additional risk factors that could further influence disease progression. Recent studies using transcriptomic techniques have identified genes involved in immune regulation, extracellular matrix remodeling, and cellular stress responses, providing valuable insights into IPF’s pathophysiology. For instance, (Maher et al., 2017; Hu and Xu, 2024) used RNA-seq and microarray analyses to reveal dysregulated gene networks implicated in IPF, including the TGF-β and Wnt signaling pathways, both of which contribute to fibrosis. However, these studies have not comprehensively explored how transcriptomic patterns correlate with IPF comorbidities, leaving potential diagnostic and therapeutic targets underexplored.

Our study addresses these research gaps by performing an integrative transcriptomic analysis of lung tissues from IPF patients. This approach not only identifies key molecular signatures associated with fibrosis but also examines the influence of comorbid conditions that may exacerbate IPF pathology. Building on previous transcriptomic studies, we aim to provide a more holistic understanding of the molecular mechanisms underlying IPF, providing insights that could facilitate the development of targeted and effective diagnostic and therapeutic interventions. Our findings contribute to IPF research by enhancing the understanding of its pathogenesis and identifying potential biomarkers that could serve as diagnostic tools or therapeutic targets, ultimately addressing some of the unsolved challenges in the field.

## 2 Methods

**FIGURE 1:**
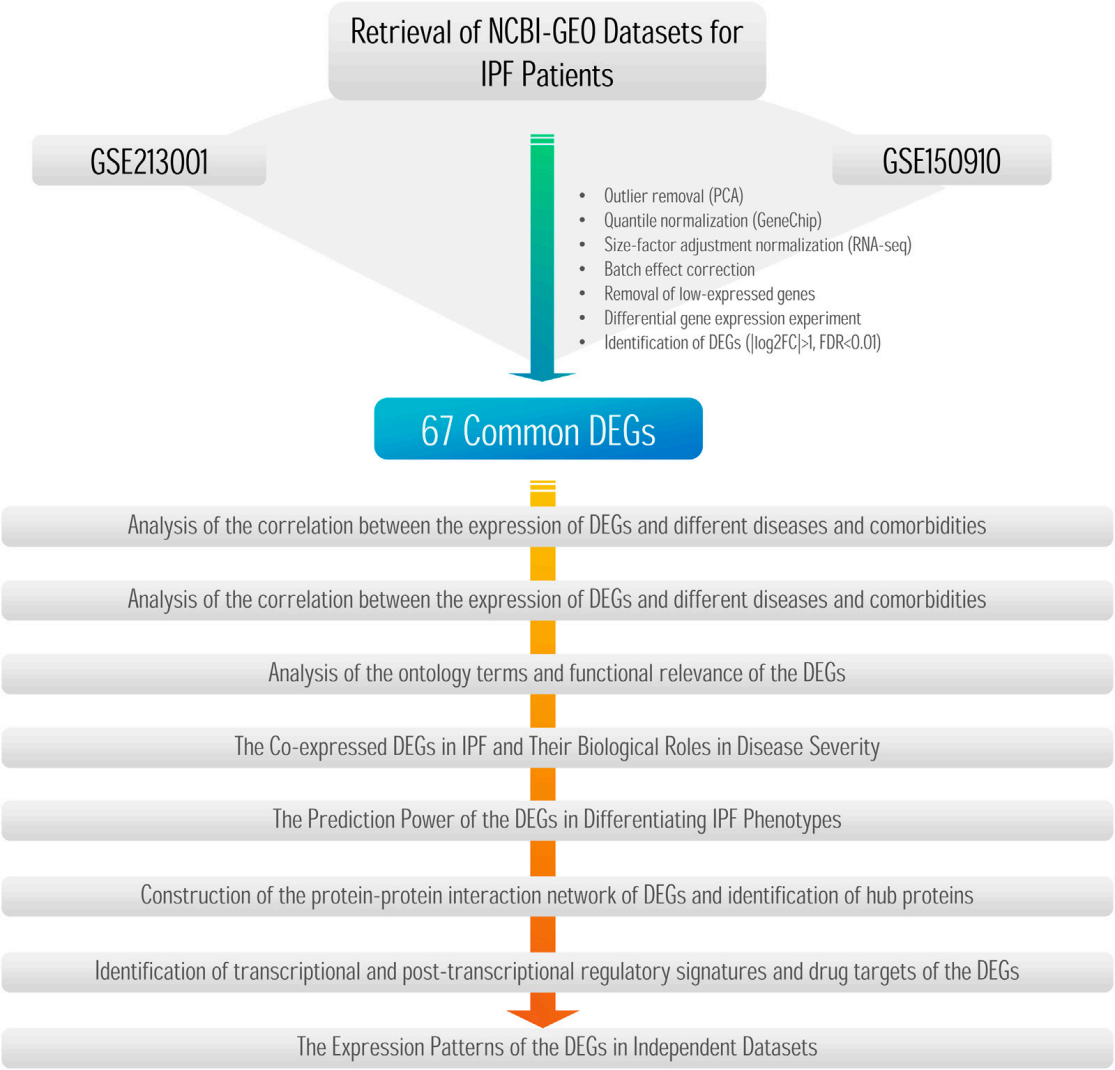
Flowchart diagram illustrating the stepwise methods employed in the study.

The methodology encompassed RNA-seq data analysis and the identification of differentially expressed genes (DEGs), along with weighted gene co-expression network analysis (WGCNA) and least absolute shrinkage and selection operator (LASSO) regression. It also included investigations of miRNA–gene interactions, transcription factors, and drug targets of DEGs, with findings validated using independent datasets. The flowchart in Figure 1 depicts the stepwise approach employed in this study.

### 2.1 Dataset selection and retrieval

A systematic search of the NCBI-GEO database was performed using keywords such as “IPF,” “idiopathic pulmonary fibrosis,” “transcriptome,” “RNA-seq,” and “lung tissue” to ensure a comprehensive selection of studies relevant to IPF and control groups. This search resulted in the selection of two datasets, namely, GSE213001 and GSE150910, based on sample size, data quality, and study relevance.

#### 2.1.1 Dataset characteristics and inclusion criteria

The GSE213001 dataset consists of RNA-seq data from lung tissue samples, including 20 IPF patients, 9 end-stage interstitial lung disease (non-IPF) patients, and 14 healthy controls. Although this dataset has a relatively small sample size for IPF, it was selected for its high-quality transcriptomic data specific to IPF (Zeng et al., 2023). To enhance statistical power, we incorporated the GSE150910 dataset, which provides RNA-seq data on 103 IPF patients, 103 healthy controls, and 82 patients with chronic hypersensitivity pneumonitis (Yu et al., 2020; Furusawa et al., 2020). Both datasets underwent quality control checks (e.g., read depth and alignment rates), and samples with evidence of treatment or culturing were excluded to avoid potential confounding effects.

#### 2.1.2 Confounding factor control

Potential confounding factors such as age, sex, race, and smoking history were controlled by integrating these variables into multivariable linear regression models. Additionally, propensity score matching was applied to balance covariates between the IPF and control groups, a standard approach in genomic studies for isolating disease impact while controlling for demographic and lifestyle factors (Rosenbaum and Rubin, 1983).

#### 2.1.3 Differential expression analysis and cutoff justification

Differential expression analysis was conducted using a false discovery rate (FDR) cutoff of 0.01 and a log_2_ fold change (|log_2_FC|) threshold of 1.5. The FDR threshold of 0.01 was selected as a more stringent alternative to the conventional 0.05 to minimize false positives, which is advantageous in large-scale transcriptomic studies (Jiang and Wong, 2009; Love et al., 2014). The log_2_FC threshold of 1.5 was chosen based on precedents in IPF research, targeting biologically meaningful gene expression changes. Smaller fold changes, although statistically significant, were considered less relevant biologically in the context of IPF, where larger shifts in gene expression are central to the disease process (Conickx et al., 2017; Zhang et al., 2019).

#### 2.1.4 Correction for multiple testing

To address the multiple testing burden associated with RNA-seq data, the Benjamini–Hochberg method was applied to control the FDR at q < 0.05, ensuring that identified DEGs remained statistically significant (Benjamini and Hochberg, 1995).

### 2.2 Analyzing the correlation between the expression of DEGs and different diseases and comorbidities

The chromosomal location of the DEGs was analyzed by submitting the overlapping DEG set from both databases as a query within the ShinyGO server (Ge et al., 2020), followed by annotation function analysis using the Metascape server to optimize the tissue-specific distribution of the DEGs and the classification of the proteins expressed by the DEG proteins (Zhou et al., 2019). Subsequently, the ggplot2 package within RStudio and the Circos online tool were used to generate visual representations of the relevant elements (Krzywinski et al., 2009; ggplot2, 2024). Protein–protein interactions (PPIs) were then analyzed by the NetworkAnalyst web-based server on the shared DEGs of lung tissues (Zhou et al., 2019). To generate the PPI network for our target genes, the integrated STRING database was utilized, considering a high-confidence cutoff threshold of >0.9 (Szklarczyk et al., 2019). Finally, to identify the nodes with the highest connectivity, a degree cutoff of 3.0 was applied. The resulting network was downloaded and further customized using Cytoscape (version 3.7.2) (Shannon et al., 2003).

### 2.3 Analyzing the correlation between the expression of DEGs and different diseases and comorbidities

A number of tools and databases were explored to investigate the association of the DEGs with respiratory tract diseases and other relevant conditions. At first, the DisGeNET plugin (within the Cytoscape tool) was used to examine the association by employing the default parameters (Piñero et al., 2017). To further evaluate the association of DEGs with the top 20 diseases, the Metascape web-utility tool and the DisGeNET online server were assessed. A p-value cutoff of 0.01 and a minimum enrichment score cutoff of 1.5 were considered to determine these associations. To gain insights into the expression patterns of DEGs in various human disease studies, the Expression Atlas server (https://www.ebi.ac.uk/gxa/home) was used (Papatheodorou et al., 2018). Moreover, to identify the known cancer-related genes in our DEG list, the Network of Cancer Genome database (http://ncg.kcl.ac.uk/) was used by keeping DEGs’ parameter settings at default (The Network of Cancer Genes, 2024). Finally, the ggplot2 package in RStudio was used to visualize the result.

### 2.4 Analysis of the ontology terms and functional relevance of the DEGs

To observe the expression patterns of the DEGs in different IPF studies, targeted genes were quarried against the Coronascape database, a repository that compiles the top 300 dysregulated genes derived from various IPF omics studies via the Metascape server (Metascape, 2024). The KEGG pathway was further analyzed using the clusterProfiler package within RStudio (Yu et al., 2012). The clusterProfiler package was also used to analyze the most prominent Gene Ontology (GO) terms such as biological processes (BPs), molecular functions (MFs), and cellular components (CCs) of the DEGs. The obtained results were subjected to a multi-test corrected p-value assessment, and subsequently, the top 15 Gene Ontology terms displaying significance were visually represented using the enrichplot and ggplot2 packages (Yu et al., 2012).

### 2.5 Co-expressed DEGs in IPF and their biological roles in disease severity

In order to determine the highly correlated gene modules and key genes based on the gene expression data, the WGCNA package of R was used, which creates a co-expression network and simplifies the interpretation of thousands of genes based on sample-to-sample similarity in expression profiles (Shi et al., 2020). First, we omitted outlier samples by using Pearson’s approach for sample clustering before constructing the co-expression network. We checked the viability of genes and samples in accordance with the WGCNA tutorial (Yin et al., 2020). Following that, we created the Pearson correlation matrix using the formula amn = |cmn|β to get the weighted adjacency matrix. Next, using the dynamic tree cut technique, all selected genes were clustered using a topological overlap matrix (TOM)-based dissimilarity measure, which divided the tree into eight modules labeled with various colors. Subsequently, the interaction between these co-expression modules was assessed using Pearson’s correlation coefficient (Yin et al., 2020). The clustering analysis revealed a hierarchical clustering of module eigen genes that summarized the modules. Based on the correlation of eigen genes, the dendrogram’s branches (the meta-modules) were categorized. In the heatmap of topological overlap, each module’s gene clusters were identified by a different color, with red denoting a positive association and blue denoting a negative correlation (Sánchez-Baizán et al., 2022).

### 2.6 Prediction power of the DEGs in differentiating IPF phenotypes

We employed binomial LASSO regression analysis of the identified DEGs from the GSE150910 dataset to predict the power of these DEGs in differentiating IPF phenotypes. LASSO regression enables a linear model between key determinants and prognostic outcomes, with variable screening and complexity correction (Weng and Ning, 2023). In addition, LASSO can filter variables and minimize model complexity without requiring large data samples, making it useful for building biological data models. To develop the prognostic key factors and prognostic outcomes model, LASSO regression was implemented in R using the glmnet package. Based on risk assessments, the sample was divided into high- and low-risk groups. To determine the model’s validity, the difference in survival time and survival status between high- and low-risk groups was evaluated. The receiver operating characteristic (ROC) curves were generated to assess the model’s accuracy (Weng and Ning, 2023).

### 2.7 Construction of the protein–protein interaction network of DEGs and the identification of hub proteins

The generic PPI network for the proteins expressed by the DEGs was constructed using the NetworkAnalyst server and STRING database (with a stringent overall confidence cutoff of 0.900) (Szklarczyk et al., 2019). Precise methods and tools, such as the Matthews correlation coefficient (MCC), global methods (edge percolated component, EPC) and closeness, were further used through the cytoHubba plugin within the Cytoscape tool to extract the top 10 most connected nodes (referred to as hub proteins) from the generic PPI network (Chin et al., 2024). Consequently, the hub proteins common to all three networks were identified and considered the most significant hubs. Afterward, the predominant biological processes associated with these overlapping hub proteins were analyzed using the clusterProfiler package in R Studio.

### 2.8 Identification of transcriptional and post-transcriptional regulatory signatures and drug targets of the DEGs

Using the NetworkAnalyst web server, the DEGs against the miRTarBase database were searched to understand experimentally validated miRNA–gene interactions (cutoff value < 3) (miRTarBase, 2024). The NetworkAnalyst tool was further used to investigate the gene–transcription factor (TF) interaction network, which drew information from the ENCODE database (cutoff value < 3) (ENCODE Project Consortium, 2011). The resulting gene–miRNA and gene–TF targets of the DEGs were then obtained and customized through the Cytoscape server. Finally, a query of the DEGs against the DrugBank database was performed to ascertain their corresponding protein targets and potential drug candidates (Wishart et al., 2018).

### 2.9 Expression patterns of the DEGs in independent datasets

The expression patterns of the DEGs found in the mainstream analysis were cross-validated with two additional independent datasets from NCBI-GEO databases, namely, GSE110147 and GSE53845, which included the transcriptome profiles of IPF and healthy lung tissues, respectively. In addition, 67 of the 68 DEGs identified in the mainstream analysis (excluding *TOGARAM2*) were found to be differentially expressed in these two datasets (Cai et al., 2018).

## 3 Results

### 3.1 DEGs in IPF and their molecular and cellular characteristics

**FIGURE 2:**
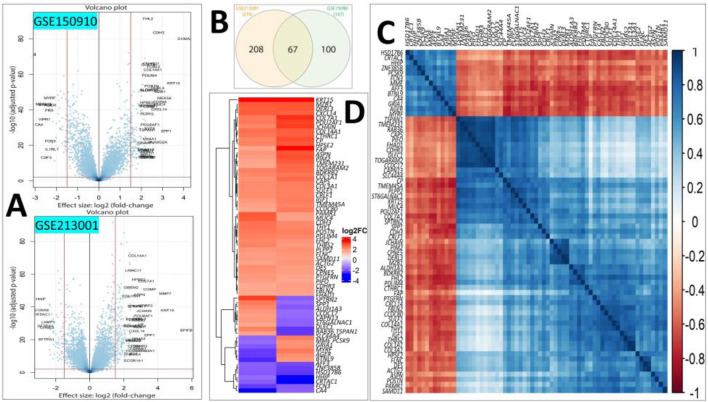
**(A)** Volcano plots showing the DEGs of our interest after filtering (|log_2_FC| > 1.6 and p < 0.01) in the GSE150910 (upper panel) and GSE213001 (lower panel) datasets. **(B)** Venn diagram showing the shared common DEGs between two datasets. **(C)** Heatmap showing the Spearman’s rank correlation among the expression values of the DEGs in IPF patients from the GSE150910 dataset. **(D)** Heatmap showing the log_2_FC values of the common DEGs in two selected datasets. The k-means clustering method was applied during both experimental procedures involved in steps **(C, D)**.

**FIGURE 3:**
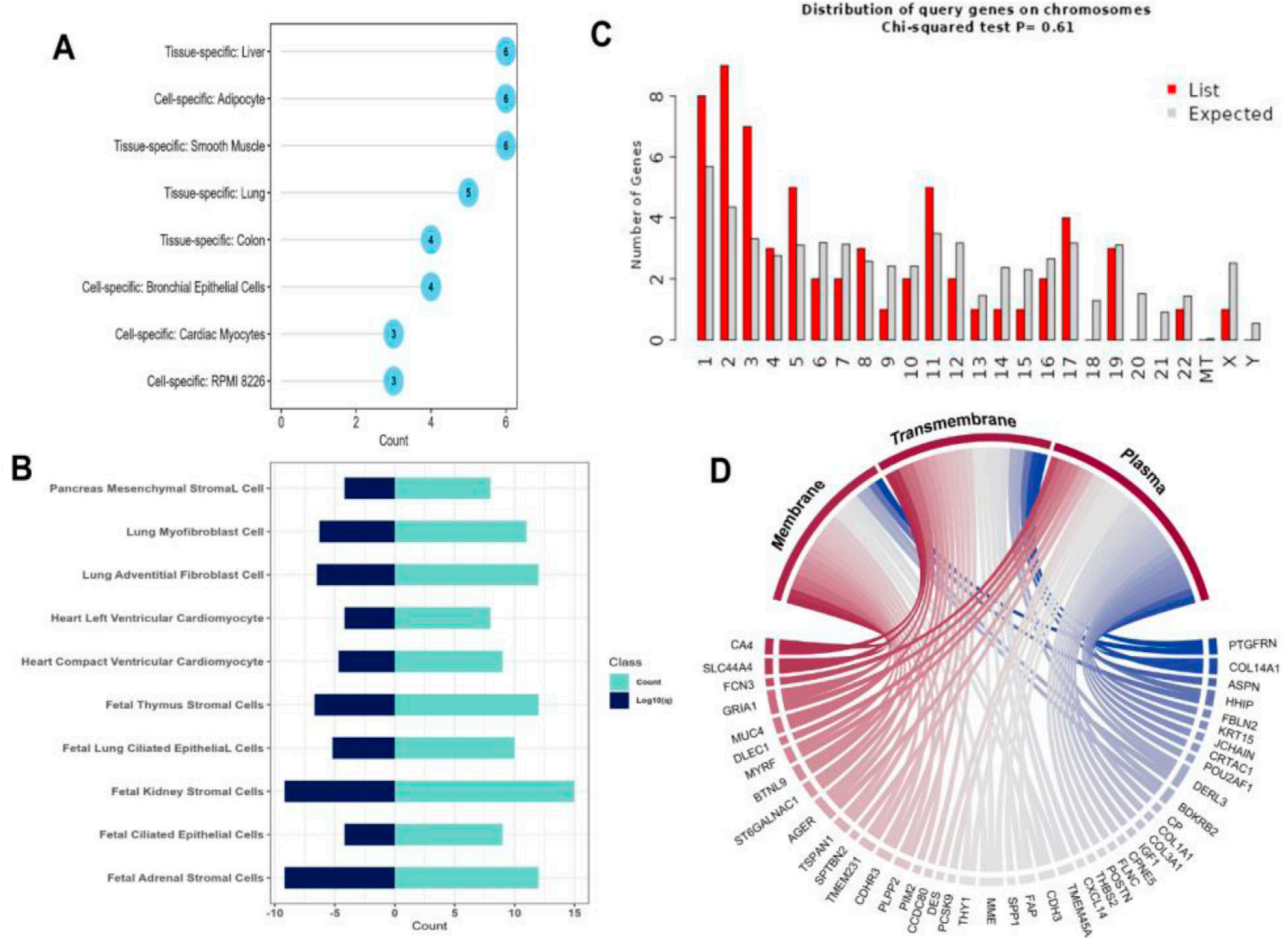
**(A)** Dot chart illustrating different tissue- and cell-specific expressions of the DEGs extracted from IPF patients. **(B)** Diverging bar-plot showing the number of genes expressed cell-specific manner. **(C)** Another bar-plot representation of the location of the DEGs identified from our experiment. **(D)** Circus plot demonstration of the functional classification of the proteins expressed by our DEGs.

Differential expression analysis revealed 275 and 167 DEGs from the GSE213001 and GSE150910 datasets, respectively (Figure 2A; Supplementary Figures S1, S2); among them, 67 DEGs are common to both datasets (Figure 2B). Considering the higher number of IPF samples in the GSE150910 dataset, a correlation analysis was performed between the DEGs among the patients’ samples from this dataset (Figure 2C). We also compared the log_2_FC of the DEGs between two datasets using a heatmap (Figure 2D). The k-means clustering in both our correlation and heatmap-based log_2_FC inspection experiment revealed that specific sets of DEGs were distinctly clustered along the plots according to their expression values (Figures 2C, D). The tissue- and cell-specific expression analysis of the DEGs revealed that five genes are expressed in lung tissues (Figure 3A), four genes are expressed in bronchial epithelial cells, and few genes are expressed in smooth muscles, adipocyte tissues, and colon and liver tissues. The cell-signature analysis of the identified DEGs in IPF patients further revealed that the DEGs are second-most expressed in lung adventitial fibroblast cells, followed by fetal thymus stromal cells and lung myoblast cells after kidney stromal cells (Figure 3B). Different types of epithelial and stromal cells were the other cell types in which our DEGs showed specific expression. Chromosomal distribution analysis of the DEGs revealed that most are located on chromosomes 1, 2, and 3. The X chromosome was found to host gene number 1. However, no gene was found to be located in the 18, 20, 21, and Y chromosomes, including the mitochondrial genome (Figure 3C). Finally, when we categorized our DEGs based on the function of their translation products, we observed that 45 genes were expressing different ranges of transmembrane, membrane, and plasma or secreted proteins (Figure 3D).

### 3.2 Disease-specific networks and expression patterns of DEGs in different disorders

**FIGURE 4:**
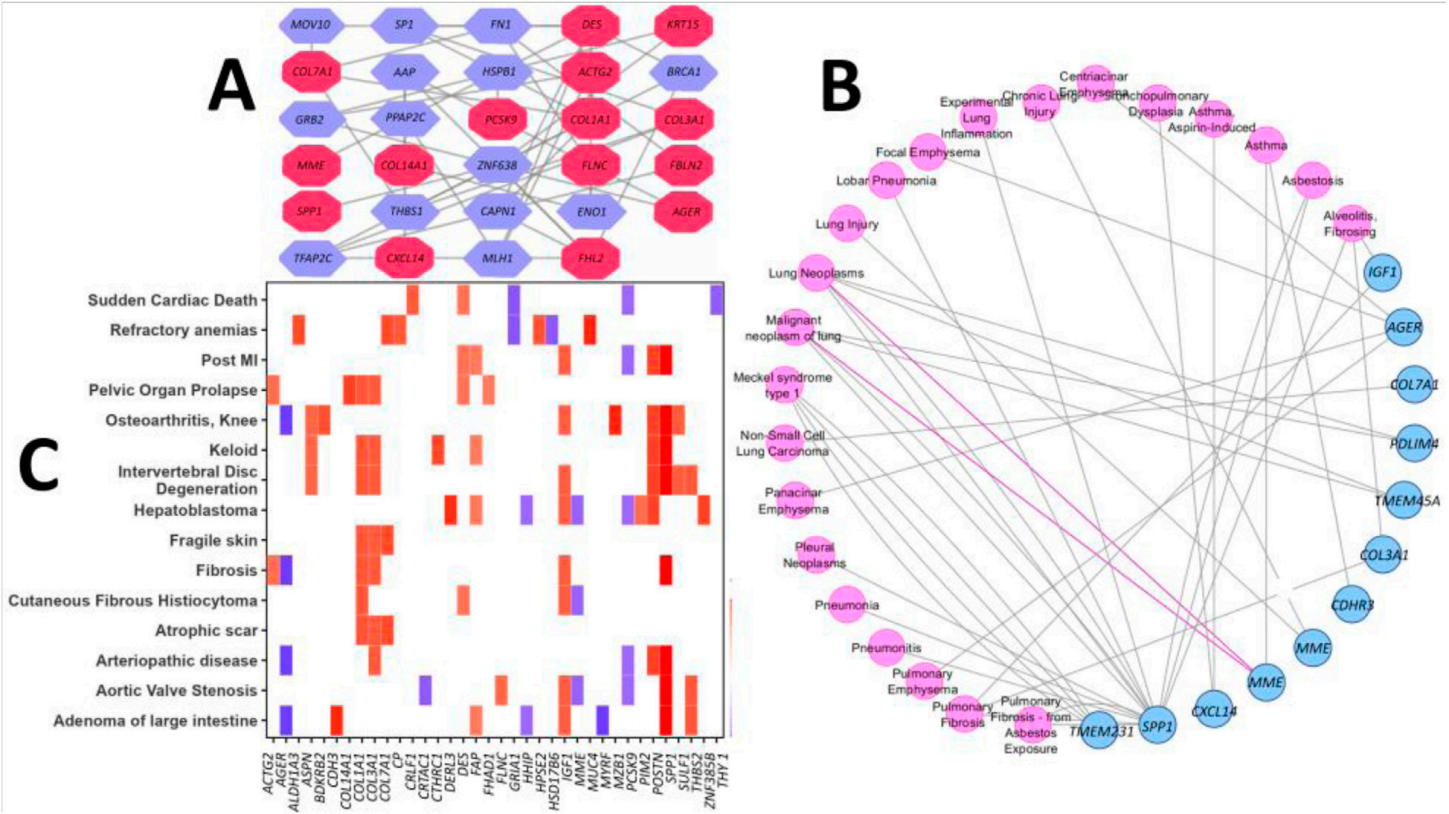
**(A)** Lung-specific PPI network of the proteins expressed by the DEGs. The red-colored nodes represent DEGs, and the remaining nodes correspond to the protein partners of the respective DEGs. **(B)** PPI network reflects the association of the DEGs with different respiratory tract diseases. Nodes represent disease term or DEGs, and edges represent connections. **(C)** Heatmap representation of the association between DEGs and different other diseases from the DisGeNET database (the log_2_FC values of the DEGs are portrayed from the GSE150910 dataset as a representative scale among the datasets selected in our study).

The lung-specific PPI network analysis revealed 15 out of 67 proteins form the interaction network with another 14 partner proteins in the lung tissues (Figure 4A). The association analysis of the DEGs in different respiratory tract diseases against curated databases of DisGeNET revealed that 12 genes of DEGs are associated with the development and progression of different lung diseases (Figure 4B). Noticeably, three genes, namely, *SPP1*, *IGF1*, and *COL3A1*, were found to be related to pulmonary fibrosis disease. Furthermore, the DEGs were found to be associated with pulmonary fibrosis from asbestos exposure, lung carcinoma, pneumonia, lung inflammation and injury, emphysema, asthma, and Meckel syndrome type 1. The disease gene-association analysis of the DEGs was performed against the curated datasets from DisGeNET, without the respiratory tract disease, to assess their association with all other types of diseases. This analysis predicted that six genes (namely, *ACTG2*, *AGER*, *COL1A1*, *COL3A1*, *IGF1*, and *SPP1*) from our DEG list were associated with fibrosis (Figure 4C). Additionally, four different genes were discovered to have associations with a type of skin fibrosis, i.e., cutaneous fibrous histiocytoma. Furthermore, other genes were differently and distinctly associated with different types of other diseases, including sudden cardiac death (Maher et al., 2021), refractory anemias (Fernández-Fabrellas et al., 2019), pelvic organ prolapse (Srour et al., 2017), atrophic scar (Issa, 2014), knee osteoarthritis (Collard, 2010), and hepatoblastoma (Sharif, 2017) (Figure 4C).

### 3.3 Functional relevance of the DEGs identified from IPF patients

**FIGURE 5:**
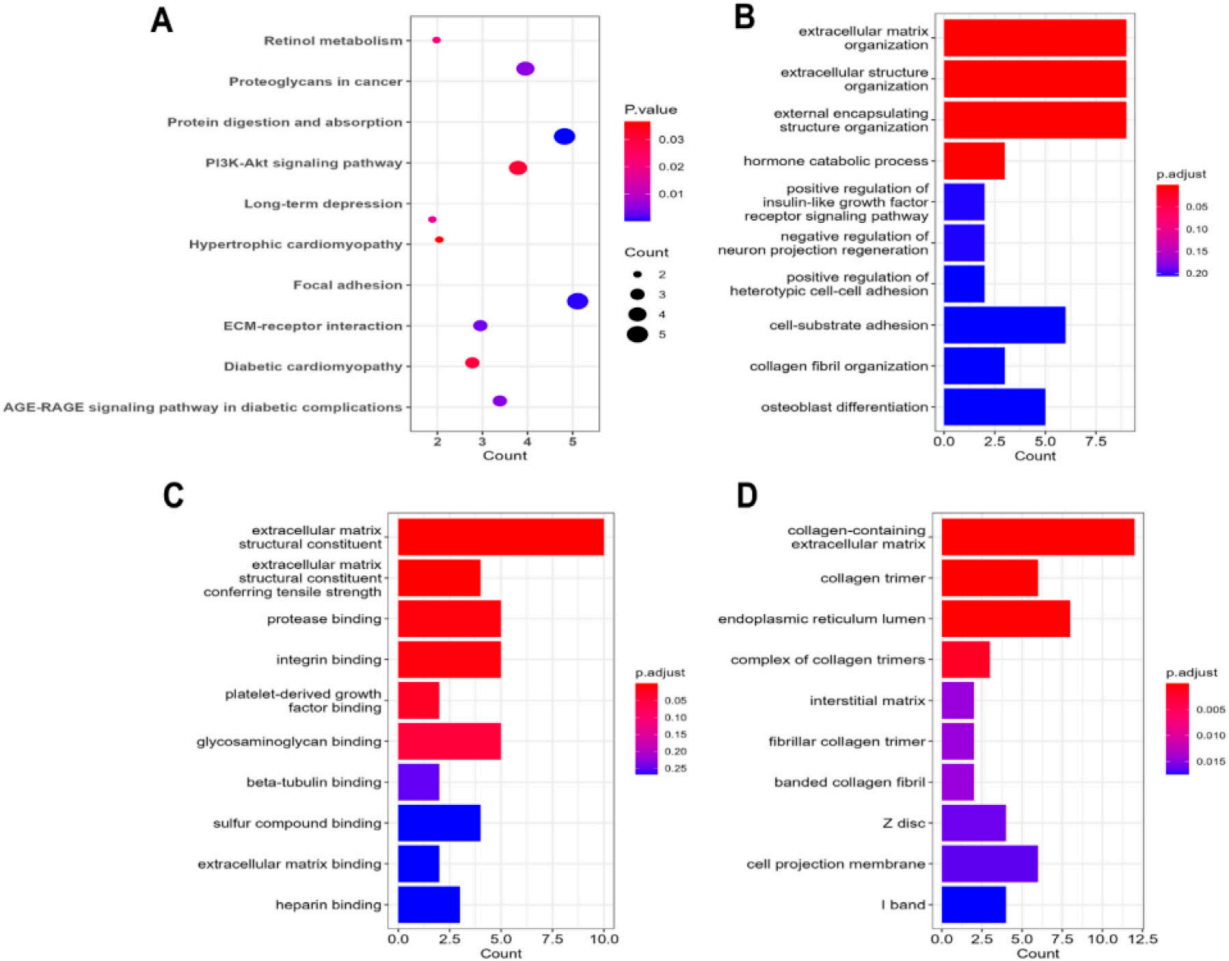
**(A)** Dot plot representation of the enriched terms obtained through KEGG pathway analysis on the common DEGs in IPF patients. The bar-plot illustration refers to the enrichment results obtained through Gene Ontology term analysis: **(B)** biological processes, **(C)** molecular functions, and **(D)** cellular components.

The KEGG pathway analysis of the common DEGs in IPF patients revealed that most of the identified DEGs were involved in the maintenance of the focal adhesion between cells (Figure 5A). The second-most largest group of DEGs in our experiments was associated with protein digestion and absorption. A notable number of the DEGs were also found to be part of the PI3K-Akt signaling pathway, AGE-RAGE signaling pathway, and ECM–receptor interactions. The analysis of GO terms found that the highest number of DEGs were significantly associated with extracellular matrix organization and maintaining its structure in terms of their major biological processes (Figure 5B). Moreover, maintaining insulin signaling pathways, hormone catabolic processes, and cell–cell adhesion were other predominant biological processes of the DEGs. On the other hand, the DEGs were predominantly involved in producing ECM constituents that provide tensile strength to the ECM, followed by protease binding, integrin binding, platelet-derived growth factor binding, glycosaminoglycan binding, and beta-tubulin binding, among others (Figure 5C). Finally, the DEGs were found to primarily function in different biological compartments, including the collagen-containing extracellular matrix, collagen trimer, complex of collagen trimmer, interstitial matrix, fibrillar collagen trimer, and banded collagen fibril, as observed through the cellular component analysis (Figure 5C).

### 3.4 Co-expressed DEGs in IPF and their biological roles in disease severity

**FIGURE 6:**
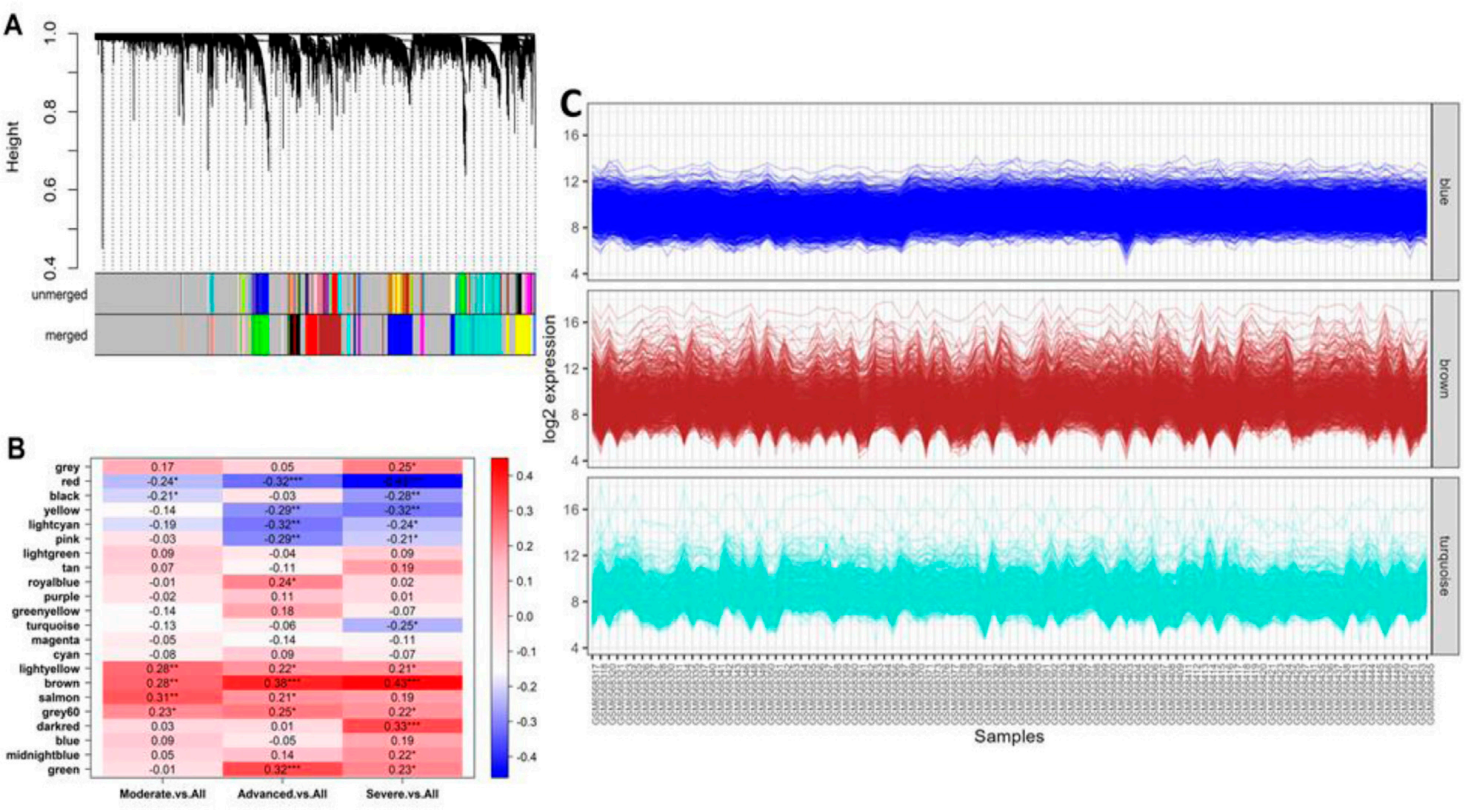
**(A)** Cluster dendrogram showing different groups of genes that were classified according to their adjacency in different modules. The clustered genes with 0.25 height in the unmerged tree were merged and incorporated into a merged tree, which was utilized in the downstream analysis. **(B)** Heatmap showing the association between different module eigen genes and different IPF severity phenotypes. The asterisks represent the level of confidence scale of the linear regression: ***, <0.001; **, <0.01; *, <0.05. **(C)** Co-expression patterns (log_2_ scale) of the genes clustered in respective best modules with a higher number of members. The brown module showed a dense co-expression pattern as it contained the highest number of genes.

The WGCNA analysis of the GSE213001 dataset resulted in a cluster dendrogram incorporating the genes in different colored modules (merged), and each module possessed dendrograms of varying heights (Figure 6A). The genes from the targeted dataset showed the highest degrees of membership with three distinct modules, namely, brown, blue, and turquoise modules, and the number of co-expressed genes in these modules ranged between 550 and 750 (data not shown here), while the brown module included the highest number of genes. Hierarchical clustering analysis of all the identified merged modules revealed that they converged into a single clade at a height of 0.9 on the tree, with no noticeable outliers, reflecting a homogenous analysis with minimal influence from technical variance (Supplementary Figure S4A). Correlation analysis between module eigen gene values (a representative scale of gene expression value) and disease severity parameters in IPF patients shows the highest correlation with IPF exacerbation. Unsurprisingly, the brown module (including the highest module members) showed a significant positive correlation (p < 0.01 and correlation: ∼0.3–0.45) with all different severity phenotypes (Figure 6B). Functional enrichment analysis of the genes that fell in the brown module reported that most of the members from this specific module are involved in crucial biological processes and molecular functions, including ECM matrix organization, collagen fibril organization, and integrin binding (Supplementary Figure S4B). We further examined the expression patterns of the shared genes in three different modules, namely, brown, blue, and turquoise, which contained the higher number of eigen genes. This analysis affirmed that the brown module included the greatest number of genes, as observed by the dense expression density in this particular module (Figure 6C).

### 3.5 Prediction power of the DEGs in differentiating IPF phenotypes

**FIGURE 7:**
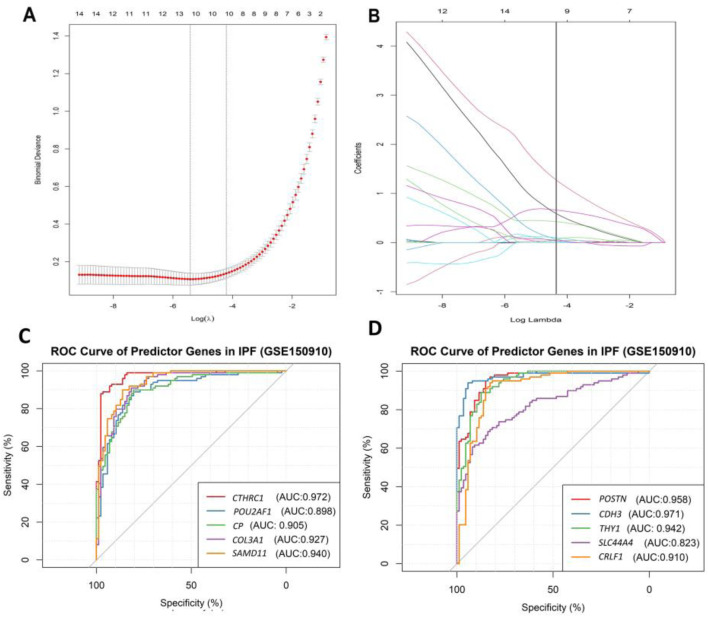
Result of binomial LASSO regression analysis on identified DEGs (GSE150910) in IPF patients. **(A)** Cross-validation curve represents different lambda values (red dotted line, presented in log scale) with upper and lower standard deviation (error bars). The vertical lines represent the lambda value with the least binomial deviance (λ._min_, left) and the lambda value with the least deviance within 1 standard error (λ._1se_, right). **(B)** Plot indicates the path of covariates in response to each lambda value. The upper axis in both plots represents the number of non-zero covariates at specific lambda values. The ROC curve of the best predictor 10 genes identified through our model: **(C)** a random group of five genes and **(D)** a random group of the remaining five genes, which was partitioned for better visualization and interpretation.

**FIGURE 8:**
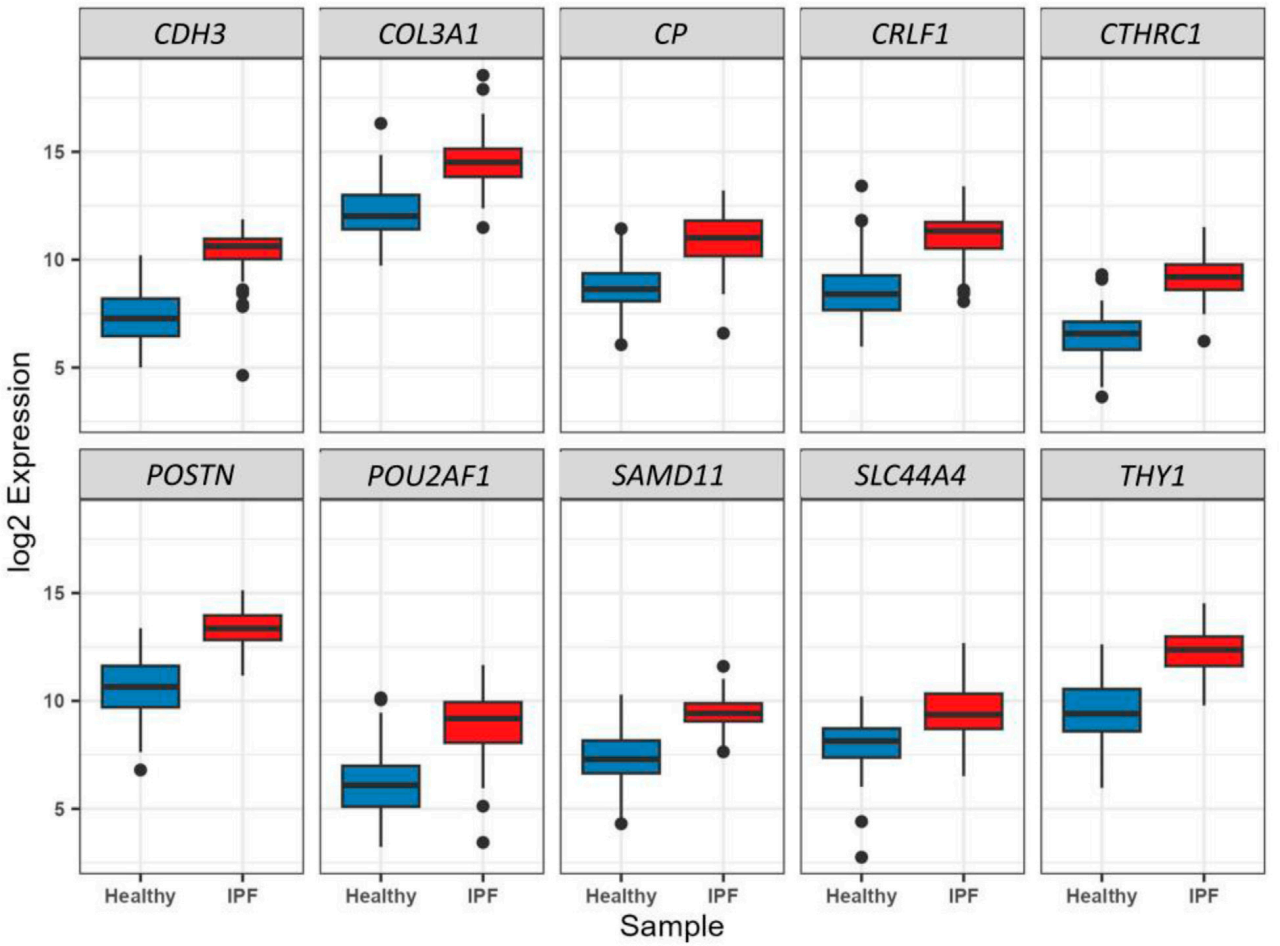
Boxplot representation of the expression difference (log_2_ scale) of the 10 selected predictor genes from LASSO regression analysis between healthy and IPF patients. All these genes were found to be significantly (p < 0.01) and differentially (log_2_FC > 1.6) expressed through Wald’s *t*-test in the primary DEG analysis (Step 1).

The binomial LASSO regression analysis of the identified DEGs from the GSE150910 dataset reported that λ._min_ from cross validation of the model was achieved at a λ value of ∼0.004, which lowered the binomial deviance to the expected threshold (Figure 7A). Using the minimum penalizer, the model retained 10 non-zero covariates (predictor genes) from a total of 67 variables (DEGs). A similar number of predictors were retained at the λ._1se_ value (one standard error from λ.min), further confirming the robustness of the model’s variable selection (Figure 7A). Inspection of the obtained model also revealed that all the covariates leave the fit model at the λ._min_ value except the non-zero predictors, as observed in the cross-validation step (Figure 7B). Thereafter, we also predicted the accuracy of our model using the ROC curve, which suggested that the proposed model has an accuracy of approximately 96.5% (Supplementary Figure S5). The 10 gene candidates that were found to be the best predictors of IPF among the 67 total DEGs were identified from our model. The area under the curve (AUC) values of these genes were assessed with ROC curves, with several genes showing exceptional predictive power. *POU2AF1* (AUC: 0.898) and *SLC44A4* (AUC: 0.823) exhibited slightly lower specificity, but their AUC values remained above 0.8, while other genes like *CTHRC1*, *POSTN*, *COL3A1*, and *CDH3* had AUC values above 0.9, indicating their excellent predictive ability (Figures 7C, D). On the other hand, the remaining genes, i.e., *CTHRC1*, *CP*, *COL3A*, *SAMD11*, *POSTN*, *CDH3*, *THY1*, and *CRLF1*, showed an AUC value above 0.9, which characterizes them as excellent predictors of IPF. Finally, we inspected the differences in the expression of these genes, which further verified the noticeable differences in their expression patterns between test and control variables (Figure 8).

### 3.6 PPI network of the proteins expressed by DEGs and their hub proteins

**FIGURE 9:**
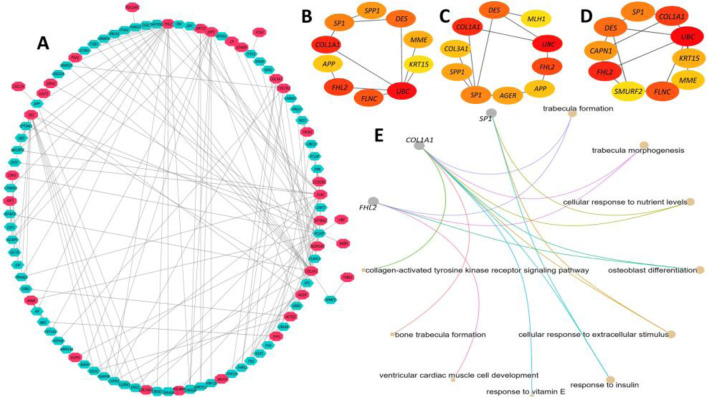
Protein-protein interaction (PPI) network analysis of the proteins expressed by the DEGs. **(A)** A PPI network was constructed using the IMEX interactome with a 1.0-degree filter, resulting in a network containing 96 nodes and 170 edges. The red-colored nodes represent proteins expressed by DEGs, while the pastel-colored nodes denote their interacting partners. The edges represent connections within the network. **(B-D)** Hub protein networks were derived from the generic PPI network using the Betweenness **(B)**, Bottleneck **(C)**, and Closeness **(D)** algorithms, each highlighting the 10 most connected nodes. Five overlapping hub proteins—SP1, COL1A1, FHL2, DES, and UBC—were identified across all three algorithms as the most significant hub proteins. **(E)** Functional analysis of the overlapping hub proteins revealed that three of them, SP1, FHL2, and COL1A1, are involved in maintaining essential biological processes in the human body. The color scale in all panels corresponds to interaction strength, with higher color density indicating stronger interactions.

Our experiment on the PPI network construction with the identified DEGs generated a PPI network with 96 nodes and 170 edges (Figure 9A). A total of 33 different proteins expressed by the DEGs were found to be part of the network. However, the interpretation of the biological relevance of the connected proteins from such a complex network often presents challenges. Hence, we further utilized the generic PPI to construct hub protein networks that represent the most connected nodes in a PPI network. The application of different algorithms, including betweenness (Figure 9B), bottleneck (Figure 9C), and closeness (Figure 9D), generated a hub network containing the top 10 most connected nodes from the generic PPI and all the networks shared by five proteins, namely, *SP1*, *COL1A1*, *FHL2*, *DES*, and *UBC* (data not provided), which are considered the most significant hub proteins from the network. Subsequent biological processes analysis of the overlapping five hub proteins indicated that three of them, namely, *SP1*, *FHL2*, and *COL1A1*, are involved in maintaining crucial biological processes inside the human body (Figure 9E). The major biological process ontology terms of these proteins included trabecula formation, trabecula morphogenesis, and response to nutrient levels, collagen-activated tyrosine kinase receptor activation signaling pathway, and osteoblast differentiation.

### 3.7 Transcriptional and post-transcriptional regulatory signatures of the DEGs

**FIGURE 10:**
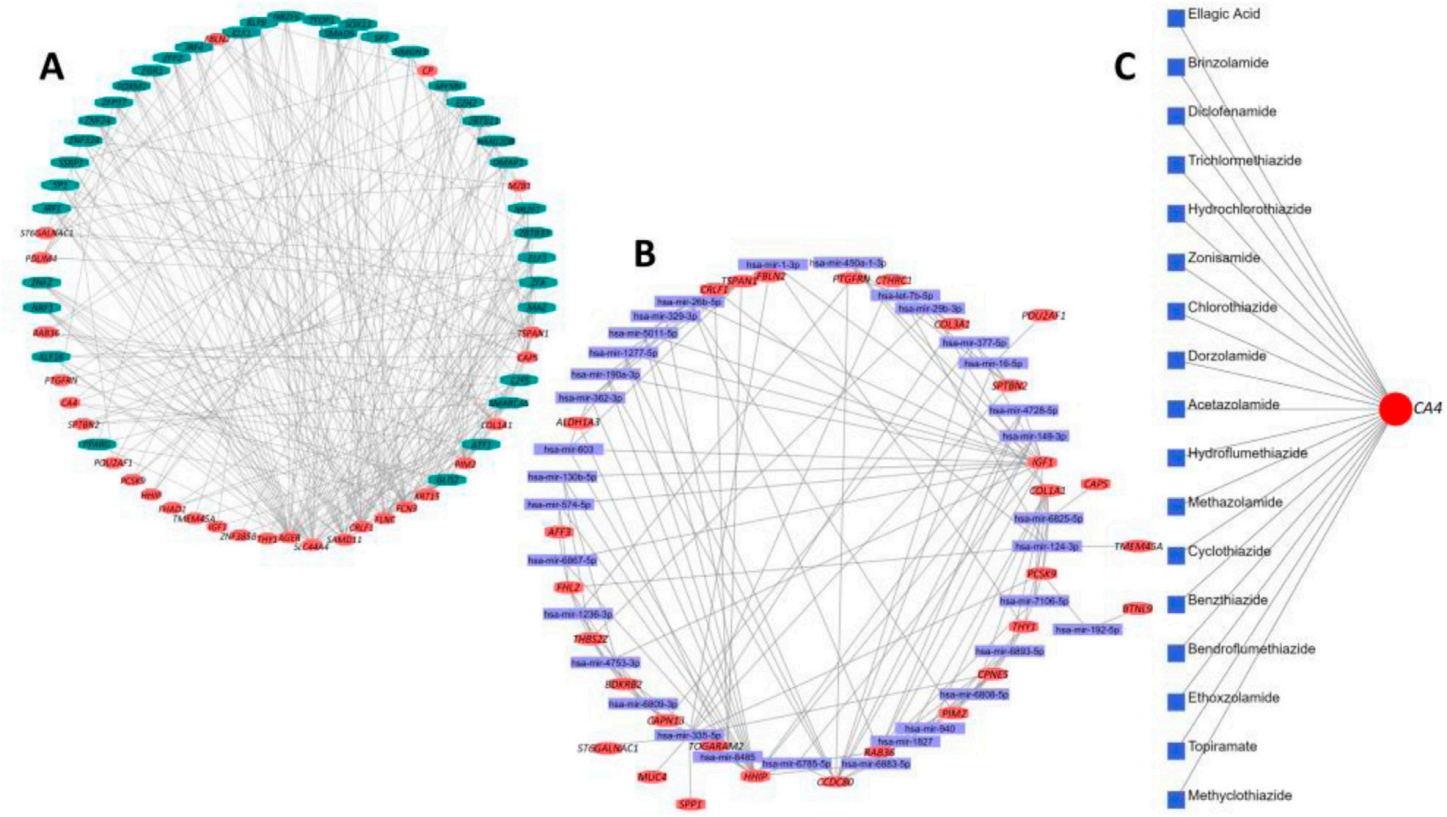
Summary of transcriptional and post-transcriptional regulator signature identification experiment on the DEGs. **(A)** Interaction network between DEGs and their respective TF partners. **(B)** Interaction network between DEGs and their respective miRNA partners. In both cases, the red nodes represent the DEGs, and the remaining nodes are their corresponding targets. **(C)** Interaction between potential drug or small candidate molecules and *CA4* gene, which was identified as a drug target for therapeutic intervention in IPF patients.

In this step, we identified the potential TFs and miRNA targets of the DEGs. The DEG–TF interaction network was found to have 64 nodes and 245 edges. In summary, 17 DEGs interacted with 49 different TFs, including *FOXM1*, *IRF4*, *EGR1*, *E2F5*, *KLF9*, *ZNF24*, *IRF1*, *SMAD5*, *NRF1*, *TFDP1*, *MAZ*, *ATF1*, *PPRAG*, *ZFP37*, *ZNF324*, *ZBTB11*, *SP7*, *EZH2*, *DMAP1*, *SOX13*, *GLIS2*, *ZLX*, and *HMGN3* (Figure 10A). On the other hand, the DEG–miRNA interaction network incorporated 63 nodes and formed 145 edges within its network (Figure 10B). A total of 30 DEGs formed interactions with 33 different miRNAs, including Hsa-mir-1-3p, Hsa-mir-6b-5p, Hsa-let-7b-5p, Hsa-mir-16-5p, Hsa-mir-26b-5p, Hsa-mir-29b-3p, Hsa-mir-124-3p, Hsa-mir-130b-5p, Hsa-mir-149-3p, Hsa-mir-192-5p, Hsa-mir-329-3p, Hsa-mir-335-5p, Hsa-mir-8485, Hsa-mir-603, Hsa-mir-940, hsa-mir-1236-3p, Hsa-mir-377-5p, and Hsa-mir-450a-1-3p. Finally, the DEG–drug interaction revealed the *CA4* gene as a potential drug target among the identified DEGs, which formed interactions with 17 small drug/candidate molecules along its network (Figure 10C). Among the selected molecules with potential therapeutic advantages, ellagic acid, brinzolamide, diclofenamide, zonisamide, hydrochlorothiazide, methazolamide, chlorothiazide, dorzolamide, acetazolamide, benzthiazide, ethoxzolamide, and topiramate were mentionable.

### 3.8 Expression patterns of the DEGs in independent datasets

Finally, the expression patterns of the DEGs identified in the main analysis were further cross-validated with two independent datasets (including the transcriptome profile of IPF and healthy lung tissues) from the NCBI-GEO databases, namely, GSE110147 and GSE53845. We found that 67 of our total 68 DEGs (excluding only *TOGARAM2*) are also significantly (FDR<0.01) and differentially expressed in these datasets (Supplementary Figure S6). Most of these genes showed a |log_2_FC| value above 1, with a fewer number of genes exhibiting a |log_2_FC| value in the range of 0.70–0.99.

## 4 Discussion

To gain deeper insights into the molecular mechanisms driving the pathogenesis and progression of IPF, we employed a comprehensive series of meta-analysis protocols to identify determinants of health risk and molecular targets in IPF patients. Together with this pooled analysis, pathway and network-based strategies provide insights into potential molecular targets for IPF (García-Campos et al., 2015). By analyzing the pattern of differential gene expression, we further understand the role and involvement of different genes in IPF (Dutta et al., 2012; Liang and Pardee, 2023).

Identifying the common DEGs between tumor and normal samples is essential for studying tumorigenesis and identifying diagnostic, prognostic, and therapeutic biomarkers. Initially, in this study, we identified 67 common DEGs using differential gene expression analysis from the GSE213001 and GSE150910 datasets. To understand their molecular and cellular characteristics, we investigated their chromosomal location and different functional compartments within the cell. Among these, 45 DEGs displayed that their translational products constitute different proportions of transmembrane, membrane, and plasma or secreted proteins, suggesting that the DEGs are involved in a diverse group of functions that drive the IPF phenotype development. This was further supported by chromosomal location analysis, suggesting that these DEGs are sporadically located across different chromosomes, affecting IPF pathogenesis in distinct ways. Notably, we identified one gene on the X chromosome with none on the Y chromosome. This suggests that male individuals inheriting the X chromosome expressing this gene may be at greater risk for developing IPF, while female individuals with two X chromosomes may be less vulnerable since the presence of a second X chromosome can mitigate the effects of a mutated one.

Later, we observed that a majority of the dysregulated genes were specifically expressed in lung tissues. Among them, four genes were specifically expressed in the bronchial epithelial cells. Transcriptomic profiling demonstrated that many upregulated genes in IPF lung tissues have minimal to no expression in normal lung tissue. Similar observations have been made regarding the presence of gene clusters with elevated expression in bronchial epithelial cells of the IPF patients compared to healthy controls (DePianto et al., 2015). Additionally, a few genes were predominantly found to be expressed in smooth muscles. Airway smooth muscle cells, as demonstrated by Carmo-Fernandes et al. (2021), contribute to the progression of lung fibrosis by expressing Wnt5a, which leads to aggravated fibrosis of the lung with poor clinical outcomes. Cell-signature analysis of the identified DEGs in IPF patients showed that these genes were the second-most expressed in lung adventitial fibroblast cells, which are the main cellular constituents of the adventitia. These fibroblasts play an essential role in regulating pulmonary vascular wall function, including the production of extracellular matrix proteins and adhesion molecules in response to vascular stresses (Stenmark et al., 2011). These results suggest that the majority of our DEGs are expressed in the lung tissues, which are the most affected in IPF, followed by lung adventitia, which has been repeatedly reported to merge into surrounding fibrotic regions (Vascular remodeling, 2024). Our DEGs also exhibited specific expression patterns in different types of epithelial and stromal cells. This finding aligns with earlier studies suggesting that altered epithelial barrier function may be implicated in the pathogenesis of IPF. Altered epithelial cells not only undergo altered morphology but also undergo changes in differentiation and function, potentially contributing to the pathogenesis of IPF (Chakraborty et al., 2022; Iyonaga et al., 1997). Plataki et al. showed that pro-apoptotic markers are upregulated in epithelial cells in IPF, which may contribute to insufficient and delayed re-epithelialization, consequently fostering fibroblast proliferation (Plataki et al., 2005). Similarly, gene expression profiles of stromal cells from patients with IPF and lung adenocarcinoma showed that several genes were differentially expressed compared to controls (Kreus et al., 2024). IPF lung exhibits substantial histological and molecular heterogeneity. Most molecular studies have heavily focused on the extensively scarred regions of the lung as these regions are typically more accessible for standard surgical biopsy (Luzina et al., 2018). Consequently, the molecular characterization of less scarred areas remains relatively unexplored. Todd et al. (2016) observed that normal-appearing lung tissue in IPF patients also exhibits the signature of lung injury, which is absent in healthy controls. Thus, it is crucial to investigate the expression profiles of genes in symptomatic and non-symptomatic lungs.

Among the identified 67 DEGs, *ASPN*, *COL1A1*, *COL3A1*, *COL14A1*, *POSTN*, and *SPP1* have been identified as hub genes for IPF previously (Zhou et al., 2019). Wan et al. (2021) proposed that these genes, along with their interplay, could influence the development of IPF by modulating IPF-related biological processes. Among these, *ASPN* expression was found to be elevated in the lungs of mouse models with pulmonary fibrosis, and its knockdown suppressed transforming growth factor-β (TGF-β)/Smad signaling and myofibroblast differentiation (Huang et al., 2022). TGF-β is a key mediator of fibrogenesis, and the upregulation of TGF-β modulates the phenotype and function of fibroblasts (Biernacka et al., 2011). Therefore, the inhibition of TGF-β is important in attenuating fibrosis. Additionally, *COL14A1* has been identified as one of the immune-related hub genes that are positively related to IPF and has shown promise as a potential biomarker for predicting IPF based on its AUC score (Fu et al., 2023). The DEGs such as *HSD17B6*, *MYRF*, and *AGER* were also found to be differentially expressed in alveolar epithelial type 1 cells from IPF lung tissues compared to healthy samples (Ghandikota et al., 2022). *HSD17B6* has been identified as the sole gene significantly upregulated in TGF-β1-treated cells and is highly expressed in mesothelial cells in IPF (Wilson et al., 2022). *MYRF* has also been identified as a potential IPF biomarker by Gao et al. (2022). Of particular significance, IPF is associated with aberrant developmental pathways, including the Hedgehog (Hh) signaling pathway (Effendi and Nagano, 2022). Given the contribution of Hh signaling to various pro-fibrotic processes, inhibiting the Hh pathway could serve as a therapeutic approach for IPF. The DEG *CXCL14* showed significantly elevated expression in lung tissues from IPF patients and in fibroblasts stimulated *in vitro* with sonic hedgehog (SHh) (CXCL, 2024). In addition, plasma levels of the CXCL14 protein were substantially higher in IPF patients than in controls but showed a considerable decrease when treated with an Hh inhibitor (CXCL, 2024; Banthien, 2024). The DEG *FHL2* is induced by TGF-β, and its overexpression significantly enhances SMAD-dependent TGF-β signaling in NIH cells, suggesting a potential role for FHL2 as a pro-fibrotic regulator in IPF (Banthien, 2024). Furthermore, suppressing *FHL2* significantly inhibits the fibrotic morphological changes in rat lung fibroblasts and primary lung fibroblasts (Shi et al., 2023). The *FHL2* inhibition effectively mitigates TGF-β1 and bleomycin-induced fibrosis processes (Shi et al., 2023). Another DEG that is upregulated by TGF-β is *SULF1*. *SULF1* is overexpressed in TGF⁃β1 induced pulmonary fibrosis in mice and human lungs compared to normal controls (Yue et al., 2011). Additionally, TGF-β serves as an important upstream regulator of the expression of another DEG, *IGF1* (Hernandez et al., 2020). The overexpression of *IGF1* has been observed in bleomycin-induced murine pulmonary fibrosis (MicroRNA, 2024) and IPF patient lung tissue (Hernandez et al., 2020). Hernandez et al. (2020) showed that knocking down IGF1 receptors in fibroblast cells resulted in a decrease in pro-fibrotic responses. Finally, the DEG *FBLN2*, which has also been reported as upregulated in patients with IPF, may serve as a potential therapeutic target for treating IPF. Zhang et al. (2023a) demonstrated that inhibiting *FBLN2* effectively suppressed the TGF-β1-induced proliferation and migration of MRC-5 cells.

The lung-specific (PPI) network analysis revealed 12 genes, including *MOV10*, *PPAP2C*, *SP1*, *APP*, and *FN1* from the DEG list, exhibiting associations with multiple protein partners. The interconnection pattern of a large number of DEGs of the PPI network signified that the DEGs may affect the functions controlled by many other genes in IPF. Notably, the transcription factor *SPP1* has also been identified as a target for gene therapy of lung fibrosis (Kum et al., 2007). The insulin-like growth factor 1 (*IGF1*)-induced activation of PI3K/Akt signaling contributes to AEC senescence, which is linked to the etiology of IPF. *COL3A1* could be a possible biomarker for monitoring the progression of IPF and non-small cell lung cancer (NSCLC) (Dong and Ma, 2017). These findings imply that the 12 DEGs engage in interactions with partner proteins, thereby influencing lung homeostasis and predisposing patients to compromised lung integrity, leading to fibrosis. Next, we examined the association of these DEGs with various respiratory tract diseases, e.g., lung inflammation, chronic lung injury, pneumonia, lung carcinoma, and emphysema. Comorbidities such as lung cancer are significantly associated with IPF-related mortality (Kreuter et al., 2016). IPF has been reported to co-exist with pulmonary emphysema and the syndrome of combined pulmonary fibrosis and emphysema (CPFE) often presents complications such as pulmonary hypertension, lung carcinoma, and acute lung injury, resulting in a poor prognosis (Cottin, 2013; Lin and Jiang, 2015). Moreover, as IPF is characterized as a form of chronic, progressive fibrosing interstitial pneumonia with an unknown etiology, the association of the IPF-related DEGs with pneumonia is not unexpected. Finally, the disease gene-association analysis on the DEGs without the respiratory tract disease filter showed that six DEGs (namely, *ACTG2*, *AGER*, *COL1A1*, *COL3A1*, *IGF1*, and *SPP1*) were associated with fibrosis. Among these, *AGER*, *COL1A1*, *COL3A1*, *IGF1*, and *SPP1*, as already mentioned, have been linked to IPF pathogenesis (Wan et al., 2021; IJMS, 2024b; Hernandez et al., 2020; MicroRNA, 2024). These genes have been implicated in liver fibrosis development as well as fibrosis development in general (Tao et al., 2018; Osganian et al., 2022; Komatsu et al., 2012). *SPP1* has also been linked to bone marrow fibrosis (Involvement, 2024) and *IGF1* in skeletal muscle fibrosis (Cells, 2024b).

Our KEGG pathway analysis revealed that many of the identified DEGs in IPF are associated with focal adhesion and protein digestion and absorption, suggesting a role in cell–cell interactions, cell adhesion, and critical signaling pathways, including PI3K-Akt and AGE-RAGE pathways. These pathways are essential for cellular processes involved in inflammation, fibrosis, and tissue repair, all of which are highly relevant to IPF.

The PI3K-Akt signaling pathway, for instance, regulates processes such as cell growth, proliferation, motility, metabolism, and survival, contributing to disease progression in IPF (Targeting PI3K, 2024). Studies have shown that the activation of PI3K/Akt leads to the overexpression of alpha-smooth muscle actin (α-SMA) in lung fibrosis and is implicated in TGF-β-induced pulmonary fibrosis (Wang et al., 2022). This overactivation contributes to the fibroblast-to-myofibroblast differentiation and excessive ECM production observed in IPF.

The AGE-RAGE signaling pathway is also significant in IPF as RAGE (receptor for advanced glycation end products) functions as a signal transduction receptor within the immunoglobulin superfamily. Reduced RAGE levels have been observed in human IPF lungs (Ohlmeier et al., 2010) and animal models of pulmonary fibrosis (Englert et al., 2011; Ramsgaard et al., 2010), highlighting its role in disease progression. AGE-RAGE signaling has been associated with increased oxidative stress and inflammatory response, contributing to the fibrotic process in IPF.

Additionally, the identification of DEGs related to ECM–receptor interactions underscores the importance of ECM remodeling in IPF. IPF is characterized by repeated cycles of tissue injury and abnormal ECM deposition due to disrupted wound healing (Walraven and Hinz, 2018). Research over the past two decades has emphasized the role of focal adhesion kinase (FAK) in fibroblast adhesion to the ECM, a critical process in fibrosis. The inhibition of FAK has been shown to reduce ECM synthesis and increase ECM degradation, thereby potentially mitigating fibrosis (Lagares and Kapoor, 2013; Dun et al., 2010).

The GO term analysis of the DEGs in IPF highlighted critical functions involved in fibrosis and inflammation, including protease binding, integrin binding, platelet-derived growth factor (PDGF) binding, and glycosaminoglycan binding. These functions play essential roles in IPF pathogenesis.

Protease binding, for instance, is crucial for activating protease-activated receptors (PARs), which mediate the effects of coagulation factors. PAR activation regulates inflammation and fibrotic responses, particularly by promoting pro-inflammatory and pro-fibrotic pathways (Lin et al., 2017). Integrin binding, particularly via integrin αvβ3, further supports this process by activating TGF-β, a key regulator of fibrosis. TGF-β signaling in IPF contributes to fibrosis progression by inducing the expression of pro-fibrotic proteins (Molecules, 2024). PDGF binding, enriched among the DEGs, suggests a role in fibroblast proliferation and migration. PDGF, produced by injured alveolar epithelial type II cells, is a critical mediator of fibroblast activation in IPF and represents an important therapeutic target (Targeting platelet, 2024). Additionally, glycosaminoglycan binding, involving ECM components such as heparan sulfate and chondroitin sulfate, regulates fibroblast activity by influencing cell migration, proliferation, and contraction. This binding impedes fibroblast recruitment, thus affecting ECM deposition and fibrosis (Jiang et al., 2010).

Our WGCNA further underscores the importance of ECM organization and collagen fibril formation in IPF. The brown module identified contains genes related to ECM functions, such as integrin binding, glycosaminoglycan binding, and growth factor binding, reinforcing the critical role of ECM remodeling in IPF progression. In IPF, abnormal ECM deposition, particularly collagen, disrupts lung architecture, contributing to fibrosis and respiratory decline. Breakdown products of ECM, generated by oxidative stress and reactive oxygen species (ROS), exacerbate fibrogenesis by stimulating inflammatory, mesenchymal, and epithelial cell responses. This highlights the potential of targeting ECM turnover and ROS-induced signaling pathways as therapeutic strategies in IPF (Kliment and Oury, 2010; Wynn and Ramalingam, 2012). FDA-approved therapies like nintedanib and pirfenidone target ECM remodeling by inhibiting collagen fibril formation and reducing fibroblast activation. Additionally, integrins, especially integrin αvβ3, have emerged as promising therapeutic targets due to their role in ECM organization and fibroblast activation. Ongoing clinical trials are investigating integrin inhibitors and RGD peptide-based therapies to disrupt fibrotic signaling (Bahudhanapati and Kass, 2017; Henderson et al., 2020).

The genes within the brown module may also serve as biomarkers for ECM turnover and fibrosis severity, offering prognostic insights into IPF progression and responsiveness to anti-fibrotic therapies. Further investigation into ECM-linked pathways could lead to more tailored and effective treatments based on ECM dynamics and integrin expression (King et al., 2011; Ley et al., 2012).

We identified 10 genes—*CDH3*, *COL3A1*, *CP*, *CRLF1*, *CTHRC1*, *POSTN*, *POU2AF1*, *SAMD11*, *SLC44A4*, *and THY1*—from a total of 67 DEGs through LASSO regression, which were found to be the best predictors of IPF. These genes hold significant promise as potential diagnostic markers for IPF. Notably, the expression of *COL3A1* and *CDH3* was higher in the lung tissues of patients with IPF compared to healthy individuals (Zhang et al., 2023b). This is consistent with previous studies that have indicated the involvement of these genes in fibrosis. Additionally*, CP* and *POSTN* have also been recognized as potential diagnostic markers for IPF (Molecules, 2024), underscoring their relevance in disease progression.

*POU2AF1*, which was found to be more highly expressed in IPF patients than in controls, is of particular interest. Knockout studies have shown that the deletion of *POU2AF1* provides protection from bleomycin-induced lung fibrosis in mice, suggesting its pivotal role in IPF pathogenesis (Li et al., 2017). In contrast, *THY1*, which is expressed in the majority of normal lung fibroblasts, is notably absent in fibroblastic foci, the characteristic lesions of IPF (Bradley et al., 2009), making it a promising marker for distinguishing active fibrotic tissue.

To further validate the diagnostic potential of these 10 genes, we performed ROC curve analysis. The results indicated that these genes collectively serve as excellent biomarkers for IPF. The reported AUC values were notably high, reflecting the robustness and accuracy of these genes in distinguishing IPF patients from healthy controls. Specifically, an AUC value approaching 1 indicates a high degree of classification accuracy, demonstrating that these markers can reliably differentiate IPF from other lung conditions. Moreover, the inclusion of *CTHRC1* and *POSTN*, both upregulated in IPF myofibroblasts, as shown in single-nucleus assays (Single-nucleus chromatin accessibility identifies, 2024), further strengthens the diagnostic capacity of this gene set.

Our interconnection pattern of the PPI network of the selected 10 DEGs signified five significant hub proteins, and targeting those proteins holds great promise as the most effective therapeutic intervention strategy for the patient group (Shen et al., 2010). Among the identified hub proteins, *SP1* could serve as a potential therapeutic target and a prognostic indicator in individuals suffering from IPF. This assertion is supported by the work of Kum et al. (Qinghua et al., 2024), which demonstrates that the inhibition of *SP1* activity at the DNA level is an effective approach for the treatment of lung fibrosis. Our findings also identify *COL1A1* as a hub protein regulated by the long-non-coding RNA H19. Through competition with miR-196a, H19 participates in the regulation of *COL1A1*, thereby mediating pulmonary fibrosis (Lu et al., 2018). Finally, the fibrosis process induced by TGF-β1 and bleomycin can be effectively reduced through the inhibition of the hub protein *FHL2* (Shi et al., 2023).

The analysis of miRNA and TF interactions revealed that multiple DEGs, along with their corresponding mRNAs, are targeted by various miRNAs and TFs. Among the identified TFs, aberrant induction of *FOXM1* has been observed in the lungs of IPF patients and mouse models of fibrotic lung injury (Balli et al., 2013). Moreover, the deletion of *FOXM1* in alveolar epithelial type II cells prevented lung fibrosis, while the overexpression of *FOXM1* in these cells exacerbated fibrosis (Balli et al., 2013). Additionally, *EGR1* is aberrantly expressed in animal models such as transgenic mice expressing TGF-ß or IL-13 and human fibrotic diseases such as IPF and scleroderma (Bhattacharyya et al., 2011). Additionally, the loss of *EGR1* protects mice from IPF, suggesting that *EGR1* may be involved in remodeling physiological and pathological connective tissue (Bhattacharyya et al., 2011). Therefore, *EGR1* presents itself as a novel pro-fibrotic mediator and holds promise as a potential target for the development of anti-fibrotic therapies. Zucker et al. (2014) demonstrated that *KLF9*, a TF identified in our analysis, independently increases the levels of ROS in cultured cells and animal tissues and is essential for the pathogenesis of bleomycin-induced pulmonary fibrosis in mice. While *NRF1* TF exerts anti-fibrotic activity in lung fibrosis through the inhibition of the TGFβ1 pathway (Suliman et al., 2022), knockdown of *NRF1* leads to increased mRNA expression of the pro-fibrotic MMP-2 and MMP-9, suggesting that upregulating Nrf1 could decrease the pro-fibrotic response of MMP-2 and MMP-9, making this TF a promising therapeutic target (IJMS, 2024a). The TF IRF4, crucial for regulating M2 macrophage polarization, exhibits overexpression in both lung sections and bronchoalveolar lavage fluid cells of IPF patients (Mou et al., 2022). We found *EZH2* as a potential target for IPF treatment. Xiao et al. reported the differential upregulation of *EZH2* in the lungs of IPF patients and mice with bleomycin-induced lung fibrosis (Wiley Online Library, 2024). Moreover, TGF-1-induced differentiation of human lung fibroblasts into myofibroblasts was reduced by *EZH2* inhibition (Wiley Online Library, 2024). Finally, many studies have shown that ATF1 influenced several fibrotic diseases, and targeting ATF1 mitigates the proliferation and activation of TGF-β-stimulated fibroblasts (MiR, 2024).

Recent evidence has highlighted the potential of multiple miRNAs as biomarkers for the early diagnosis of IPF. In our study, hsa-let-7b-5p, hsa-miR-29b-3p, and hsa-miR-26b-5p were identified as hub miRNAs, consistent with their roles as diagnostic biomarkers in IPF, as also reported in previous studies (He et al., 2022). These miRNAs are involved in critical processes such as ECM deposition, TGF-β signaling, and immune modulation, which are central to the pathogenesis of IPF. Notably, our study provides additional validation of their involvement in IPF-specific pathways. Earlier studies, including ours, have identified other key miRNAs, such as hsa-miR-16-5p, hsa-miR-26b-5p, hsa-miR-335-5p, hsa-miR-124-3p, and hsa-miR-192-5p, as the most relevant post-transcriptional signatures in IPF (Catalanotto et al., 2016). Among these, exosomal miR-142-3p has been shown to attenuate fibrosis in airway epithelial cells by inhibiting the TGF-β signaling pathway, indicating its anti-fibrotic effect in IPF (Guiot et al., 2020). The miRNome analysis by Granata et al. further emphasized the significance of miR-8485 as an upregulated miRNA in bronchial epithelial cells, specifically in the context of everolimus-induced pulmonary fibrosis (Granata et al., 2018).

Finally, we cross-validated the results using two independent datasets from the NCBI-GEO databases, namely, GSE110147 and GSE53845, which comprise the blood transcriptome profiles of IPF and healthy lung tissues. We found that 67 out of 68 (excluding *TOGARAM2*) are differentially expressed in the blood cells of IPF patients, which holds the potential for aiding in the development of non-invasive diagnostic approaches for this patient population. The consistency of the observed expression changes across multiple datasets strengthens the robustness and reliability of our findings. Furthermore, the majority of the DEGs exhibited a fold change (log2FC) above 1, indicating substantial differences in expression between the IPF and healthy lung tissues. This suggests that these genes may play important roles in the development or progression of IPF as their expression levels are significantly altered compared to healthy samples.

Although our study provides valuable insights into IPF-specific DEGs, certain limitations should be acknowledged. Potential biases may arise from the dataset selection, which might not fully capture inter-individual variability, and from analysis techniques that could influence DEG detection and interpretation. Despite these limitations, the identified biomarkers hold strong translational potential, offering promising candidates for future diagnostic tools and targeted therapies for IPF. By advancing our understanding of IPF-related molecular pathways, these findings pave the way for personalized treatment strategies aimed at improving patient outcomes.

## 5 Conclusion

This study investigates the molecular basis of IPF, pinpointing 67 key genes linked to the disease, with a focus on notable genes like *ASPN* and *COL1A1*. It also identifies potential therapeutic targets and regulators through protein interactions, microRNAs, and transcription factors. Ten genes are identified as strong diagnostic markers for IPF through LASSO regression. Gene module analysis provides insights into the biological processes contributing to IPF severity. The consistency of findings across independent datasets strengthens the reliability of these results, suggesting their utility for non-invasive diagnostic approaches in IPF. This study paves the way for future research and clinical applications, advancing our understanding of IPF and potentially leading to more targeted and effective diagnostic and therapeutic strategies for this challenging disease.

## Data Availability

The original contributions presented in the study are included in the article/Supplementary Material; further inquiries can be directed to the corresponding author.
