# Supplementary material for: Identifying health risk determinants and molecular targets in patients with idiopathic pulmonary fibrosis via combined differential and weighted gene co-expression analysis
Source: Front Genet. 2025 Jan 29;15:1496462. doi: 10.3389/fgene.2024.1496462 (PMC11813903; doi:10.3389/fgene.2024.1496462)
Supplement: Supplementary file 1 [file Table1.DOCX]

**Supplementary Information**


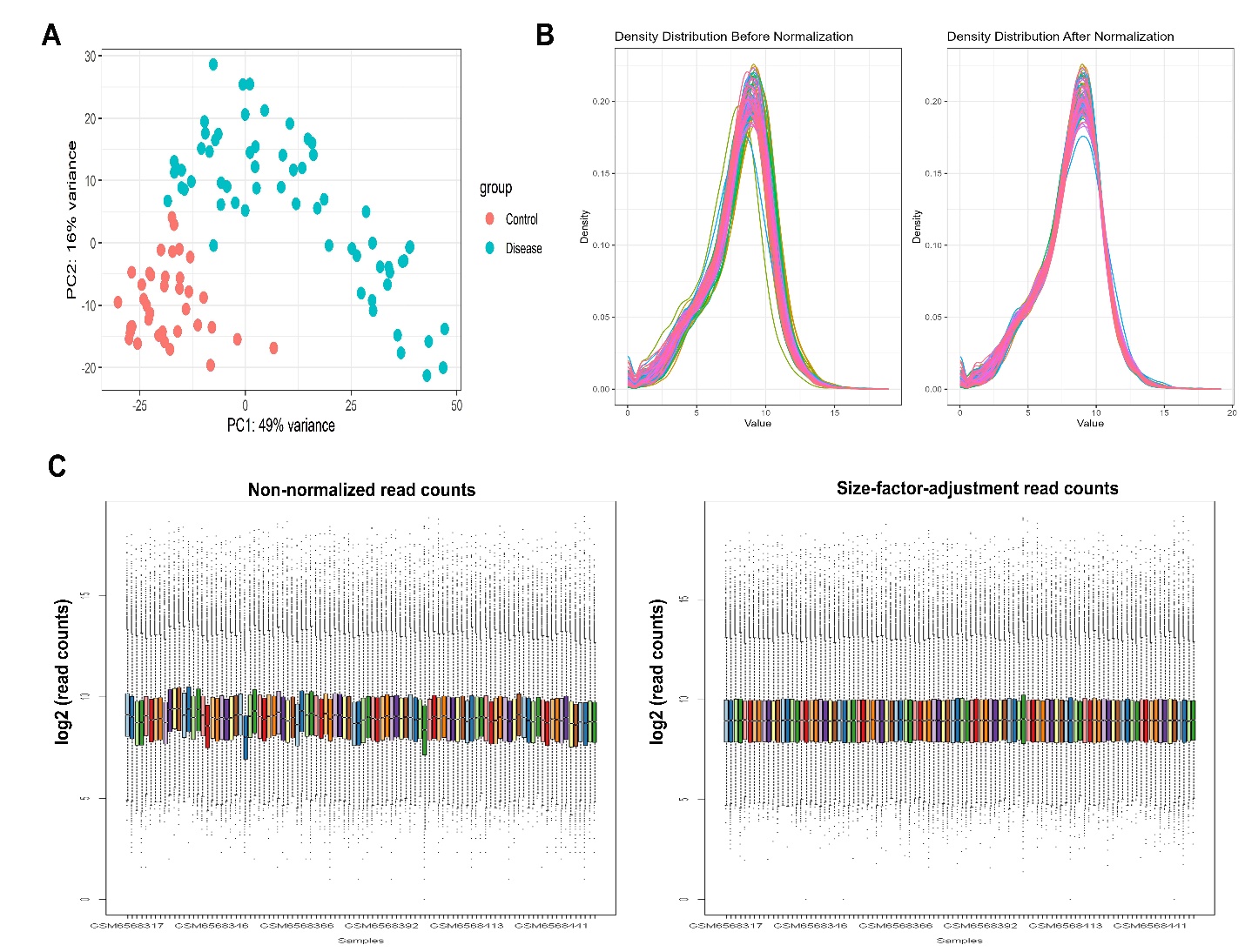


**Supplementary Figure S1:** The quality assessment report on the GSE213001 dataset. (A) PCA plot representing that the two variables (i.e., control and disease) were distinctly clustered upon dimensionality reduction. (B) The skewness in the density distribution of the expression values after normalization reflects the effect of batch-effect removal while retaining the biological variances. (C) The boxplot representation of the effect of size-factor-adjustment normalization on the average expression of samples that reduces the between-sample deviances in the selected cohort.


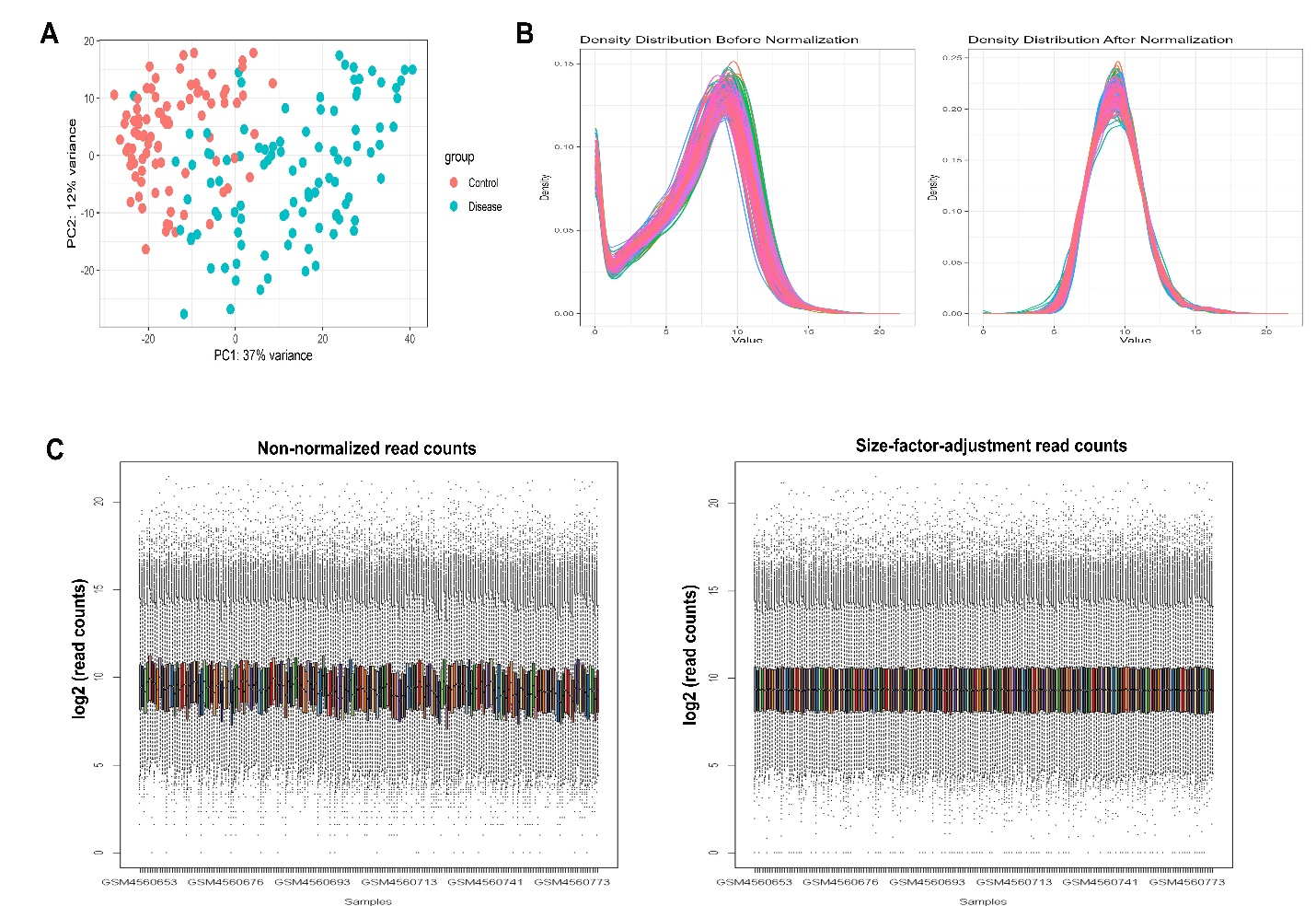


**Supplementary Figure S2:** The quality assessment report on the GSE150910 dataset. (A) PCA plot representing that the two variables (i.e., control and disease) were distinctly clustered upon dimensionality reduction. A slight overlapping among a few samples was observed even after removing outliers might be due to the presence of IPF patients with varying degrees of severity. (B) The skewness in the density distribution of the expression values after normalization reflects the effect of batch-effect removal while retaining the biological variances. (C) The boxplot representation of the effect of size-factor-adjustment normalization on the average expression of samples that reduces the between-sample deviances in the selected cohort.


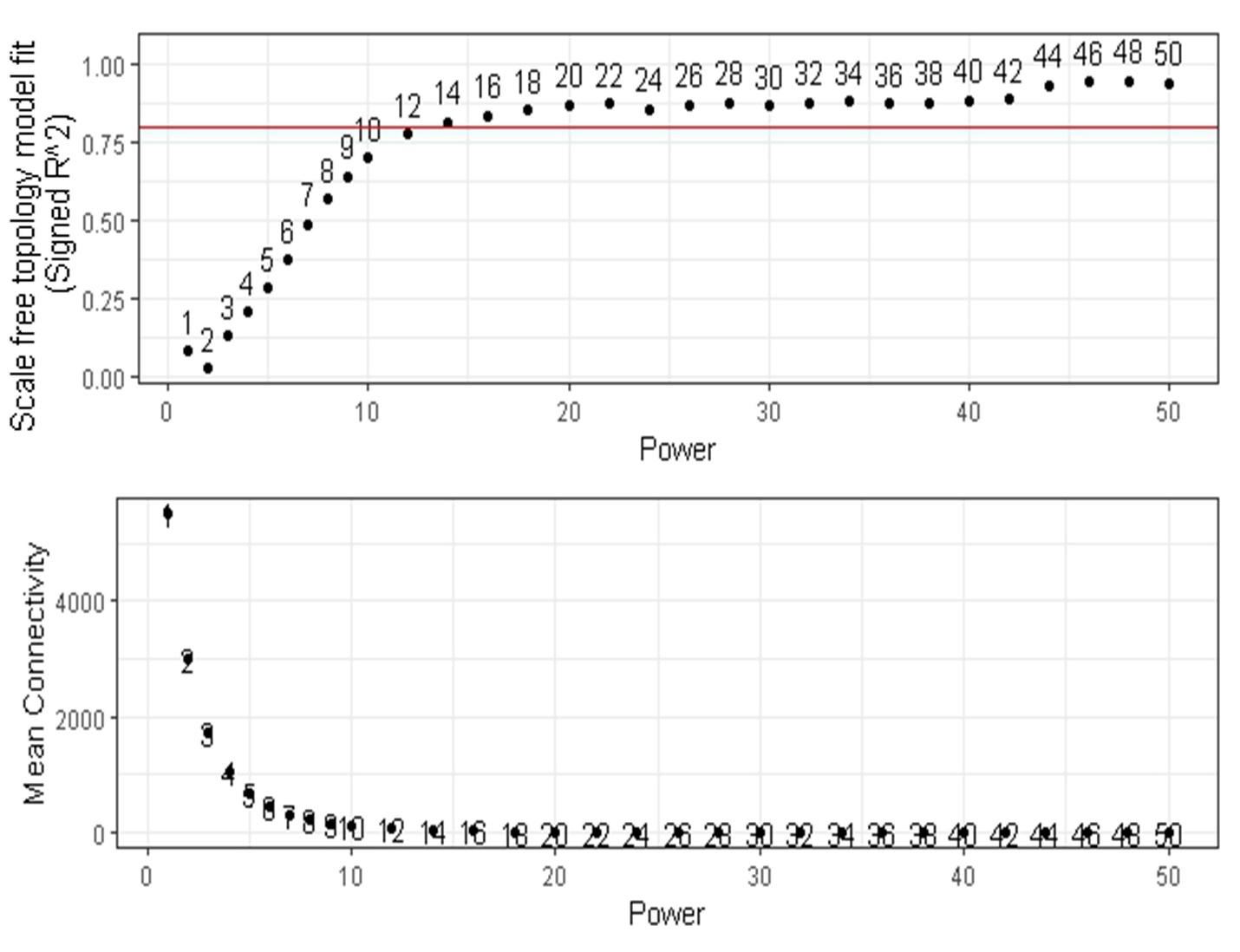


**Supplementary Figure S3:** The plots show the scale-free topology fit at different soft-thresholding power (upper panel). The expected scale-free topology fit (above 0.8, R^2^) is achieved at a power of 14 which was used to generate the cluster modules. The mean connectivity also falls down to a lower value (less than 100) at 14 power value which merely varies with a slight increase or decrease of the soft threshold power (lower panel).


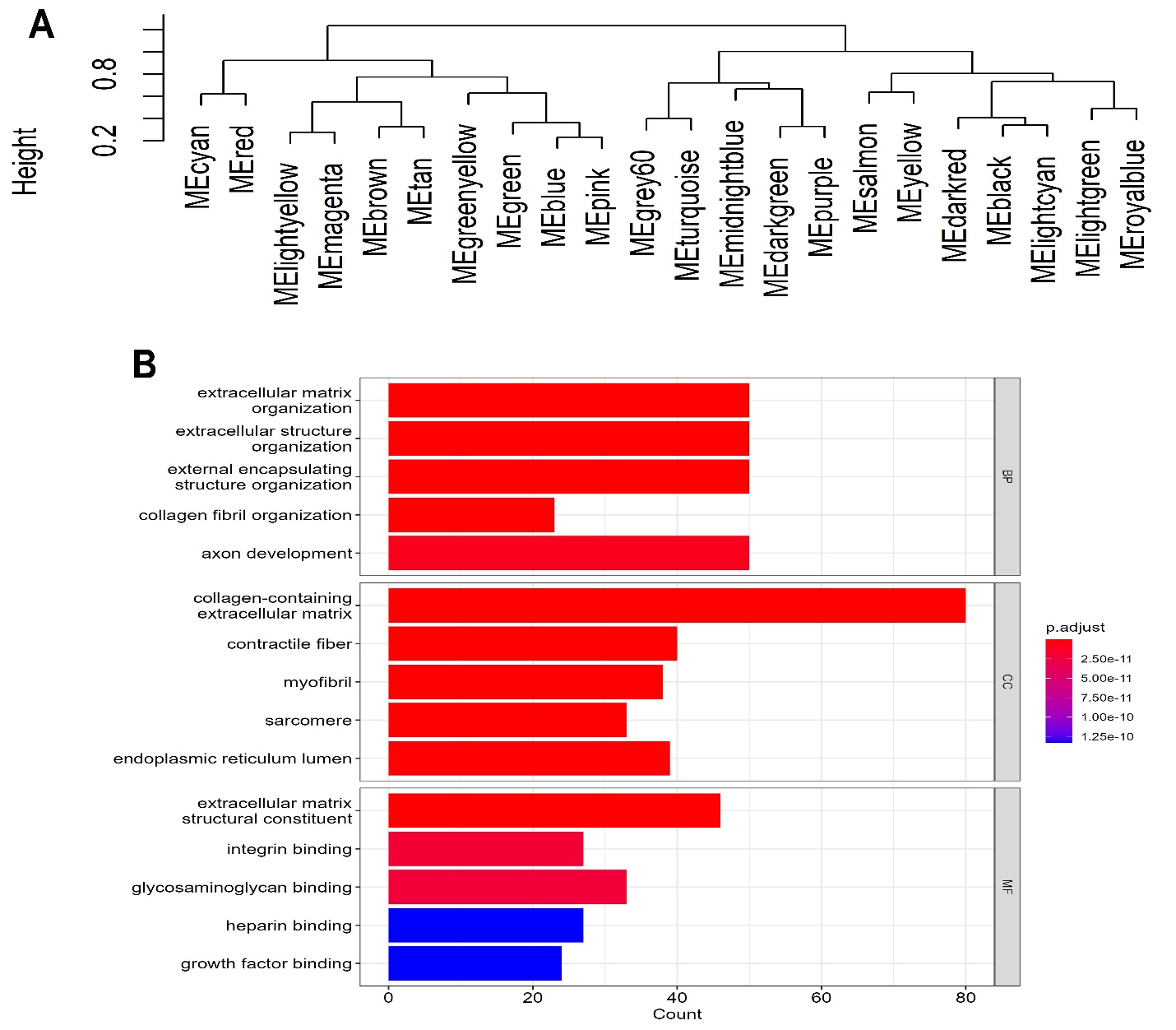


**Supplementary Figure S4:** (A) The hierarchical clustering of the identified modules from weighted gene co-expression analysis (WGCNA) that resulted in two homogenous major clusters containing multiple members in each cluster. (B) The results of the gene ontology term analysis on the brown module genes from WGCNA analysis. BP: biological processes, MF: molecular function, CC: cellular component.


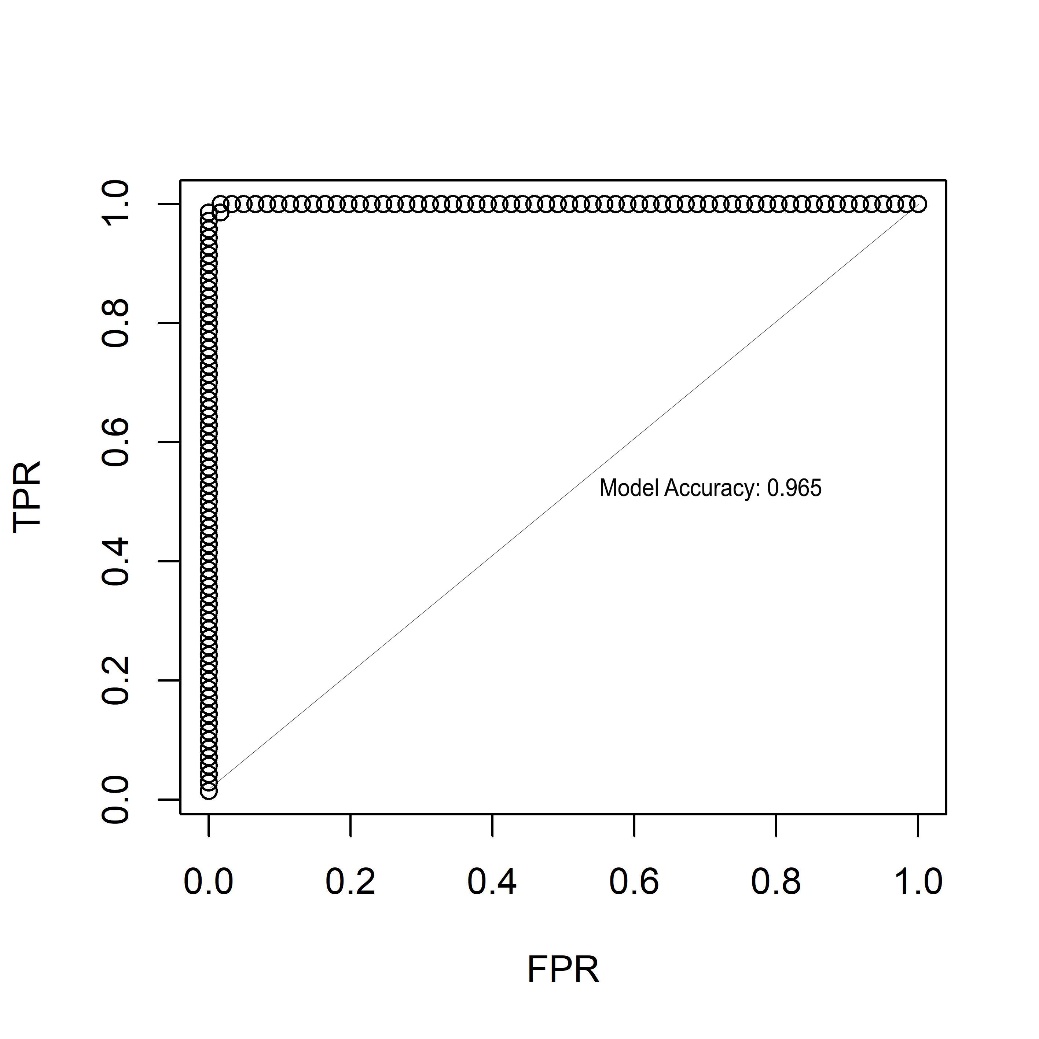


**Supplementary Figure S5:** The receiver operating characteristic curve on our LASSO regression model fit that was predicted to have 96.5% accuracy. TPR: true positive rate; FPR: false positive rate.


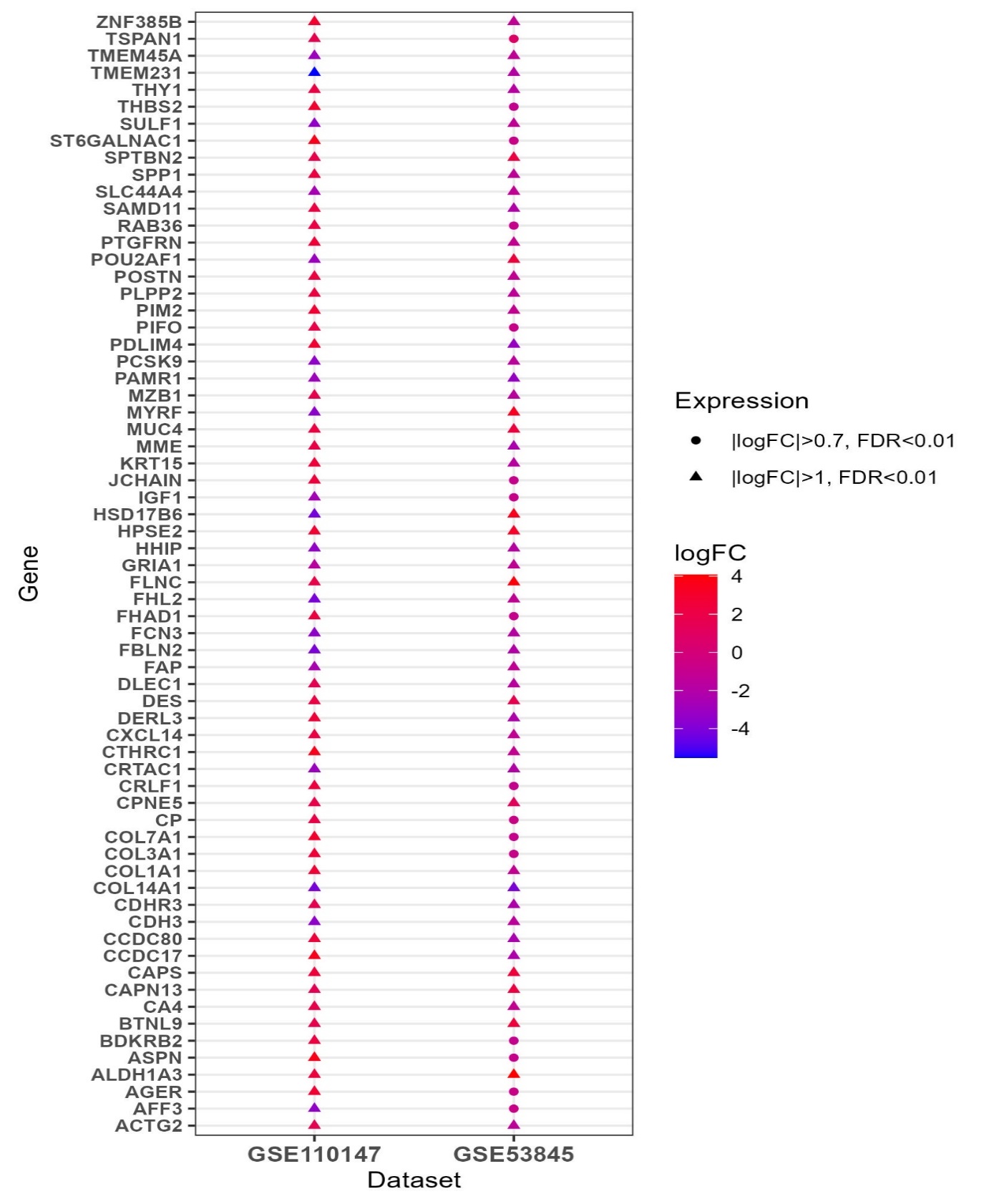


**Supplementary Figure S6:** The result of expression analysis on the identified DEGs from GSE110147 and GSE53845 datasets. Among 68 DEGs from mainstream analysis, 67 (except *TOGARAM2*) were found to be differentially expressed in these two datasets as well.
