# Supplementary material for: Identifying health risk determinants and molecular targets in patients with idiopathic pulmonary fibrosis via combined differential and weighted gene co-expression analysis
Source: Front Genet. 2025 Jan 29;15:1496462. doi: 10.3389/fgene.2024.1496462 (PMC11813903; doi:10.3389/fgene.2024.1496462)
Supplement: Supplementary file 2 [file Presentation1.pptx]

## Slide 1
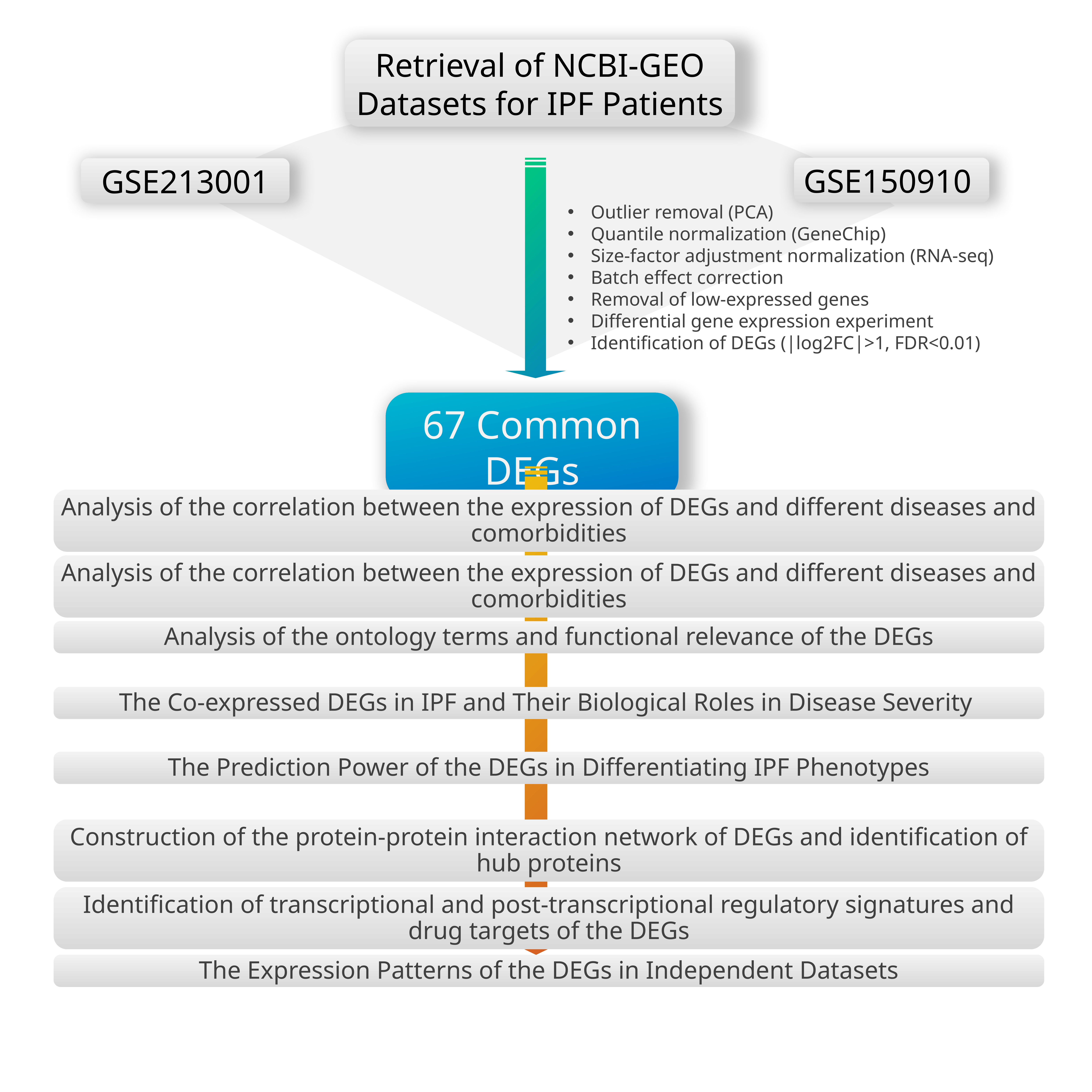

Retrieval of NCBI-GEO Datasets for IPF Patients
GSE150910
GSE213001
Outlier removal (PCA)
Quantile normalization (GeneChip)
Size-factor adjustment normalization (RNA-seq)
Batch effect correction
Removal of low-expressed genes
Differential gene expression experiment
Identification of DEGs (|log2FC|>1, FDR<0.01)
67 Common DEGs
Analysis of the correlation between the expression of DEGs and different diseases and comorbidities
Analysis of the correlation between the expression of DEGs and different diseases and comorbidities
Analysis of the ontology terms and functional relevance of the DEGs
The Co-expressed DEGs in IPF and Their Biological Roles in Disease Severity
The Prediction Power of the DEGs in Differentiating IPF Phenotypes
Construction of the protein-protein interaction network of DEGs and identification of hub proteins
Identification of transcriptional and post-transcriptional regulatory signatures and drug targets of the DEGs
The Expression Patterns of the DEGs in Independent Datasets
